# Mapping the influence of hydrocarbons mixture on molecular mechanisms, involved in breast and lung neoplasms: in silico toxicogenomic data-mining

**DOI:** 10.1186/s41021-024-00310-y

**Published:** 2024-07-09

**Authors:** A’edah Abu-Bakar, Maihani Ismail, M. Zaqrul Ieman Zulkifli, Nur Aini Sofiyya Zaini, Nur Izzah Abd Shukor, Sarahani Harun, Salmaan Hussain Inayat-Hussain

**Affiliations:** 1 Product Stewardship and Toxicology, Environment, Social Performance & Product Stewardship (ESPPS), Group Health, Safety and Environment (GHSE), Petroliam Nasional Berhad (PETRONAS), Kuala Lumpur, 50088 Malaysia; 2 Health, Safety and Environment (HSE), KLCC Urusharta, Kuala Lumpur, 50088 Malaysia; 3 Institute of Systems Biology, Universiti Kebangsaan Malaysia, Bangi, Selangor 43600 UKM Malaysia; 4 ESPPS, GHSE, PETRONAS, Kuala Lumpur, 50088 Malaysia; 5 Department of Environmental Health Sciences, Yale School of Public Health, Yale University, 60 College St, New Haven, CT 06250 USA

**Keywords:** Breast cancer, Lung cancer, Hydrocarbons, Toxicogenomics Analysis, Chemical mixtures, Molecular pathways

## Abstract

**Background:**

Exposure to chemical mixtures inherent in air pollution, has been shown to be associated with the risk of breast and lung cancers. However, studies on the molecular mechanisms of exposure to a mixture of these pollutants, such as hydrocarbons, in the development of breast and lung cancers are scarce. We utilized in silico toxicogenomic analysis to elucidate the molecular pathways linked to both cancers that are influenced by exposure to a mixture of selected hydrocarbons. The Comparative Toxicogenomics Database and Cytoscape software were used for data mining and visualization.

**Results:**

Twenty-five hydrocarbons, common in air pollution with carcinogenicity classification of 1 A/B or 2 (known/presumed or suspected human carcinogen), were divided into three groups: alkanes and alkenes, halogenated hydrocarbons, and polyaromatic hydrocarbons. The in silico data-mining revealed 87 and 44 genes commonly interacted with most of the investigated hydrocarbons are linked to breast and lung cancer, respectively. The dominant interactions among the common genes are co-expression, physical interaction, genetic interaction, co-localization, and interaction in shared protein domains. Among these genes, only 16 are common in the development of both cancers. Benzo(a)pyrene and tetrachlorodibenzodioxin interacted with all 16 genes. The molecular pathways potentially affected by the investigated hydrocarbons include aryl hydrocarbon receptor, chemical carcinogenesis, ferroptosis, fluid shear stress and atherosclerosis, interleukin 17 signaling pathway, lipid and atherosclerosis, NRF2 pathway, and oxidative stress response.

**Conclusions:**

Within the inherent limitations of in silico toxicogenomics tools, we elucidated the molecular pathways associated with breast and lung cancer development potentially affected by hydrocarbons mixture. Our findings indicate adaptive responses to oxidative stress and inflammatory damages are instrumental in the development of both cancers. Additionally, ferroptosis—a non-apoptotic programmed cell death driven by lipid peroxidation and iron homeostasis—was identified as a new player in these responses. Finally, AHR potential involvement in modulating *IL-8*, a critical gene that mediates breast cancer invasion and metastasis to the lungs, was also highlighted. A deeper understanding of the interplay between genes associated with these pathways, and other survival signaling pathways identified in this study, will provide invaluable knowledge in assessing the risk of inhalation exposure to hydrocarbons mixture. The findings offer insights into future in vivo and in vitro laboratory investigations that focus on inhalation exposure to the hydrocarbons mixture.

**Supplementary Information:**

The online version contains supplementary material available at 10.1186/s41021-024-00310-y.

## Introduction

Air pollution, a pervasive mixture of chemicals and particulate matter (PM), is one of the greatest environmental risks to health. In 2019, the World Health Organization (WHO) estimated 11% of outdoor air pollution-related premature deaths were due to cancer within the respiratory tract [1].

Polycyclic aromatic hydrocarbons (PAHs) are among the chemicals found in the complex mixture of chemicals and PM in air pollution [2, 3]. Common sources of PAHs include household combustion devices, motor vehicles, industrial activities, and forest fires [2]. Exposure to airborne PAHs in both occupational and non-occupational settings were associated with the risk of developing breast and lung cancers [2–8]. Notably, a French prospective cohort study, of a large sample size with long-term exposure data of benzo(a)pyrene (BaP), showed significant association between airborne BaP exposure and overall breast cancer risk. The association was greater among women in menopausal transition and tobacco smokers [3]. Inevitably, the International Agency for Research on Cancer (IARC) classified BaP as a Group 1 carcinogen in humans, based on sufficient experimental evidence of carcinogenicity in animals and corroborated by consistent mechanistic evidence [9].

The IARC has also declared tobacco smoking to have sufficient and limited evidence in humans to cause lung and breast cancer, respectively [10]. Arguably, tobacco smoking is a good example of adverse health effects of exposure to chemicals mixture. This is because tobacco smoke contains more than 5,000 different chemicals, including PAHs, tobacco specific nitrosamines, aromatic amines, aldehydes, phenols, nitro compounds, volatile hydrocarbons, and other organic and inorganic chemicals [11]. Tobacco smokers who work at industrial facilities are at high risk of exposure to hydrocarbons mixture and the risks of breast and lung cancers have been shown to be greater among workers who smoke tobacco [3, 12]. Studies on the mechanism by which exposure to a mixture of hydrocarbons contributes to the development of breast and lung cancers are scarce and, indeed, a complex field to venture into. However, advances in toxicogenomics provide comprehensive databases on chemicals, genes, proteins, and diseases that one can utilize to gain insights into molecular pathways that chemical mixtures potentially influence in the development of a specific disease.

This article elucidates interactions of genes influenced by a mixture of carcinogenic hydrocarbons with those related to the development of breast and lung cancer. Importantly, the article demonstrates the capability of in silico data-mining for gauging probable molecular mechanisms of mixture-induced toxic effects. This may then assist in strategizing experimental studies to better understand the impact of airborne hydrocarbons in the development of breast and lung cancers. The findings of such studies would then contribute to the risk assessment of chemical mixtures to safeguard the health of people.

## Methods

### Selection of hazardous air pollutants

In 2019, Ismail et al. [13] undertook to prioritize the hazard classification of 188 chemicals in the Office of Environment Health Hazard Assessment (OEHHA) list of chemicals emitted from California refineries [14]. The prioritization was in accordance with the United Nations Globally Harmonized System of Classification and Labelling of Chemicals (UN GHS). The classifications considered were carcinogenicity (C), mutagenicity (M) and reproductive toxicity (R) from databases of nine countries. Out of the 188 chemicals, 67 were identified as carcinogens 1 A (known human carcinogen), 1B (presumed human carcinogen) or 2 (suspected human carcinogen) [13].

We confirmed the classification of these chemicals by referencing databases of six countries—Australia, European Union (EU), Japan, South Korea, Malaysia, and New Zealand—to reflect the latest classification. The reference databases (Table 1) were chosen as they were accessible in English on the open World Wide Web domain.

**Table 1:** Source of the UN GHS classification database

Governmental Agency	Status of chemical list	Source
Japan	Advisory	https://www.nite.go.jp/en/chem/chrip/chrip_search/srhInput
Malaysia	Regulatory	(https://dosh.gov.my)
Australia	Advisory	http://hcis.safeworkaustralia.gov.au
New Zealand	Regulatory	https://www.epa.govt.nz/database-search/chemical-classification-and-information-database-ccid/
European Chemicals Agency	Regulatory	https://echa.europa.eu/
South Korea Ministry of Environment	Regulatory	http://ncis.nier.go.kr/en/main.do

From the revised list, chemicals with the most stringent carcinogenicity classification (1/1A/1B) (Suppl Table 1) were then screened for hydrocarbons, as they are common air pollutants and contained in tobacco smoke. These hydrocarbons were further analyzed for gene interactions in the development of breast and lung cancers. The molecular pathways potentially influenced by these genes were elucidated to gain insights on potential molecular pathways affected by hydrocarbons mixture.

### Comparative Toxicogenomic database (CTD) analysis

**Fig. 1:**
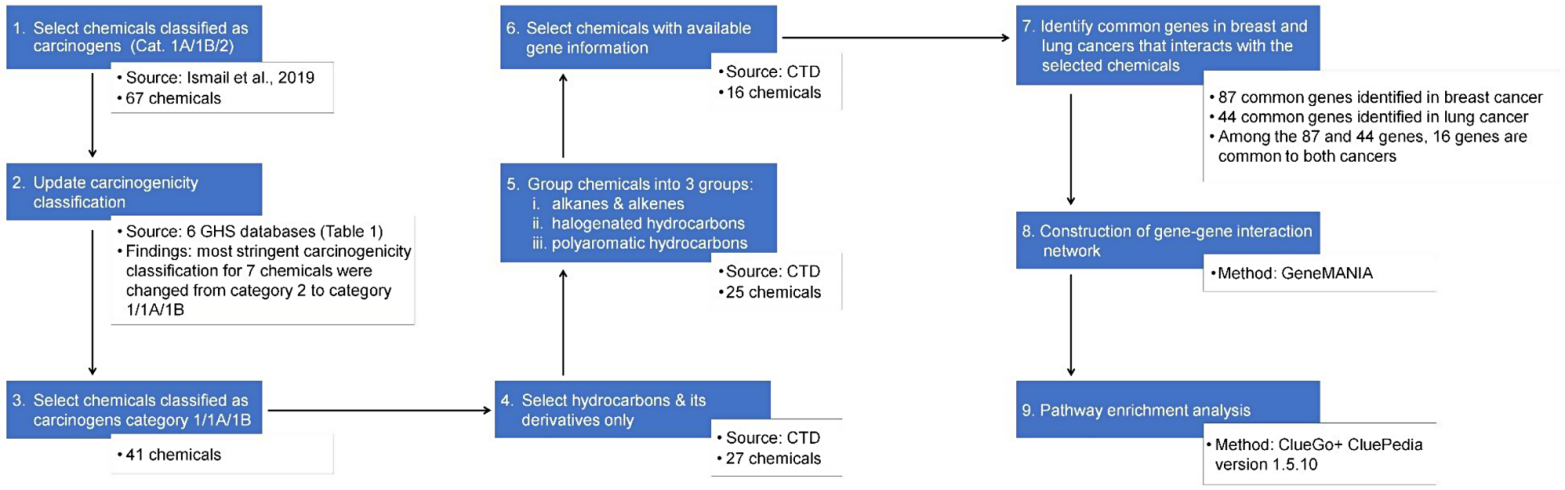
Process flow for *in-silico* data-mining

The hydrocarbons were grouped into alkanes/alkenes, halogenated hydrocarbons, and PAHs. The linkages between these groups of hydrocarbons and cancers of the breast and lung, were explored by analyzing the chemical-gene/protein interactions obtained from the Comparative Toxicogenomic Database (CTD; https://ctdbase.org/). The analysis was based on data downloaded in July 2023. The CTD is a public domain database that allows the integration of data to provide a better understanding of the interactions between environmental chemicals, genes, and diseases [15]. Chemicals, chemical-phenotypes, gene ontology and chemicals-disease associations are the examples of information provided by the CTD. The search for genes associated with breast and or lung cancers was based on the CAS number of each individual carcinogenic hydrocarbon and inference network. The data-mining process flow is depicted in Fig. 1. The respective inference score and the reference links are in Supplementary Table 2.

### Identifying common genes for hydrocarbons mixture and breast and lung cancer development

The lists of genes extracted from the CTD were uploaded to an Excel spreadsheet. Further analysis was done with Cytoscape version 2.5.10—a free software package—to visualize, model and analyze molecular and genetic interaction networks [16].

### Gene-gene interaction network construction

The complex gene-gene interactions network of the common genes between the hydrocarbons and the selected cancers was constructed with GeneMANIA, a free in silico tool (http://www.genemania.org) that provides a flexible interface to query genomic, proteomic, and gene function data [17, 18]. The tools’ dataset are from various publicly available databases, such as Gene Expression Omnibus (GEO) for co-expression data [19]; BioGRID for physical and genetic interaction data [20]; I2D for predicted protein interaction data [21]; and Pathway Commons for pathway and molecular interaction data [22–25]. The database has almost 2300 networks from eight different organisms that collectively contain nearly 600 million interactions covering almost 164,000 genes [18]. GeneMANIA generates networks from the data either directly or using an in-house analysis pipeline to convert profiles to functional association networks [26]. Co-expression networks were filtered (by default) to remove weak correlations [18]. In this study, *Homo sapiens* was selected as a target organism in GeneMANIA analysis.

### Molecular pathways enrichment analysis

**Fig. 2:**
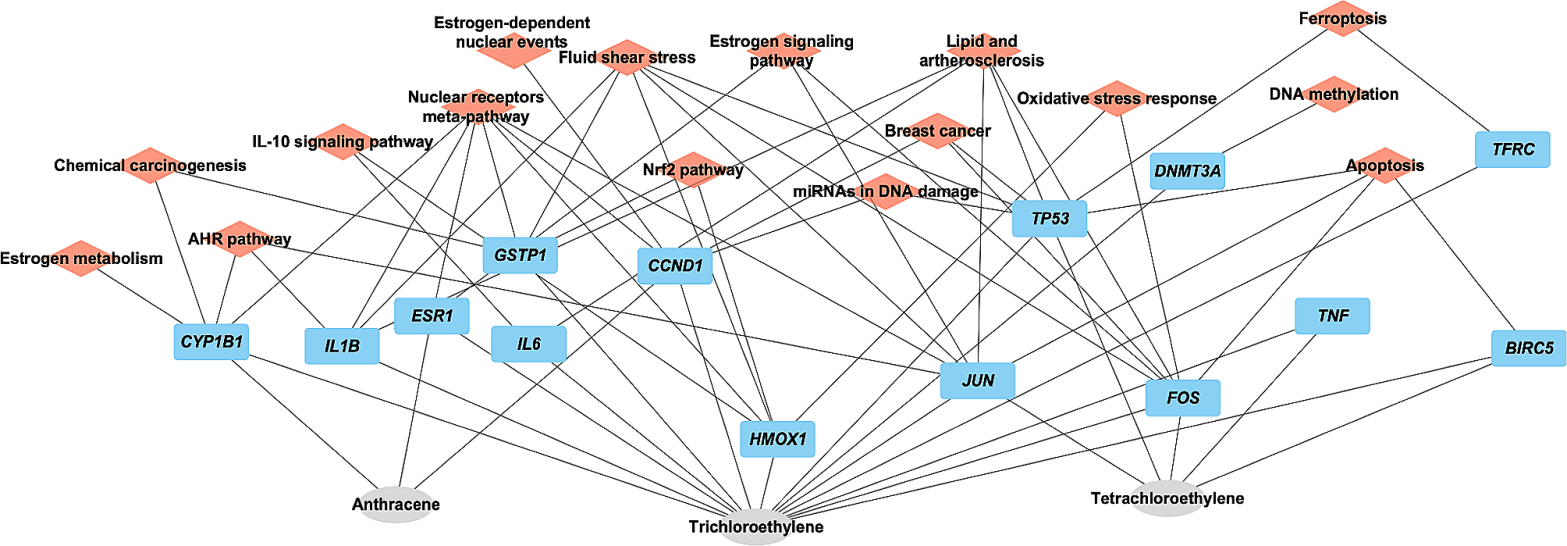
Gene and molecular pathway interactions of hydrocarbons associated with breast cancer

**Fig. 3:**
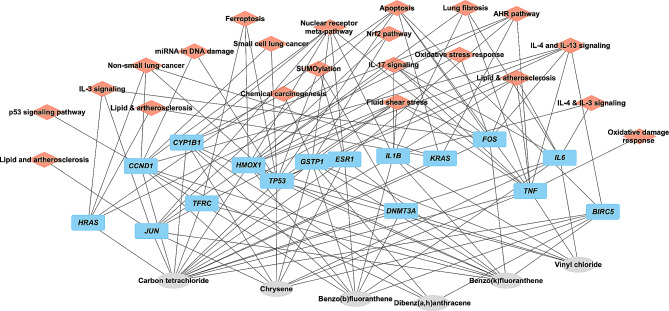
Gene and molecular pathway interactions of hydrocarbons associated with lung cancer

Pathway analysis was performed by Cytoscape ClueGO together with CluePedia plug-in version 2.5.10. The common genes found between hydrocarbons that are associated with the selected cancer development were inserted into the Load Marker List section. The Kyoto Encyclopedia of Genes and Genomes (KEGG), Reactome, and WikiPathways [27–29] databases were selected in the ClueGO settings to extract the list of pathways. Enrichment right-sided hypergeometric test was used for the enrichment with a Bonferroni step-down correction and a κ score of 0.3 to link the terms [30]. ClueGO plug-in integrates GO terms and KEGG/BioCarta pathways. The plug-in was used to visualize molecular pathways and gene ontology that are linked to the examined common genes connected to the selected cancers. The organism analyzed was set to *Homo sapiens*. The output results (gene-pathway interactions) are shown in Supplementary Figs. 1 & 2 that were used to construct Figs. 2 and 3.

## Results

### Classification revision

Among the 67 chemicals screened, the classifications of seven chemicals were revised to a more stringent category: from category 2 (suspected human carcinogen) to category 1/1A/1B (known human carcinogen/presumed human carcinogen). This revision was based on Japan’s most stringent classification. The seven chemicals are 1,1,2,2-tetrachloroethane (CAS No. 79-34-5), analine (CAS No. 62-53-3), anthracene (CAS No. 120-12-7), biphenyl (CAS No. 92-52-4), dichloromethane (CAS No. 75-09-2), methyl isobutyl ketone (CAS No. 108-10-1), and styrene (CAS No. 100-42-5) (Suppl Table 1). With the revision, 41 of the 67 chemicals are category 1/1A/1B carcinogens, of which 27 are hydrocarbons (Suppl Table 1). Among the 27 hydrocarbons, 25 were identified as alkanes, alkenes, halogenated hydrocarbons, and PAHs.

### Comparative Toxicogenomic database analysis

Among the 25 hydrocarbons, nine were excluded from further toxicogenomics analysis for absence of curated data in the CTD. The nine chemicals are isobutane (CAS No. 75-28-5), n-butane (CAS No. 106-97-8), 1,1,2,2-tetrachloroethane (CAS No. 79-34-5), 1,2-dichloroethane (CAS No. 107-06-2), vinyl bromide (CAS No. 593-60-2), polychlorinated biphenyl (CAS No. 1336-36-3), benz(a)anthracene (CAS No. 56-55-3), biphenyl (CAS No. 92-52-4), and benzo(e)pyrene (CAS No. 192-97-2) (Suppl Table 2).

In the alkanes/alkenes group, only two of the four chemicals contained gene interactions data. Isoprene (2-methyl-1,3-butadiene (CAS No. 78-79-5)) affects 12 and 8 genes associated with breast and lung cancer, respectively, whilst 1,3-butadiene (CAS No. 106-99-0) affects 63 genes linked to breast cancer development and 48 genes linked to lung cancer (Suppl Table 2).

In the halogenated group, six of the twelve chemicals affect genes linked to breast or lung cancer genes. Tetrachlorodibenzodioxin (TCDD) (CAS No. 1746-01-6) interacts with the greatest number of genes related to breast cancer development (489 genes), followed by trichloroethylene (CAS No. 79-01-6; 230 genes), tetrachloroethylene (CAS No. 127-18-4; 40 genes), and dichloromethane (CAS No. 75-09-2; 35 genes). Both 1,2 dichloropropane (CAS No. 78-87-5) and 1,2-dibromoethane (CAS No. 106-93-4) affect only five genes (Suppl Table 2). Regarding genes linked to lung cancer development, TCDD, carbon tetrachloride (CAS No. 56-23-5), dichloromethane, and vinyl chloride (CAS No. 75-01-4) affect 250, 184, 25, and 16 genes, respectively, whilst 1,2-dichloropropane and 1,2-dibromoethane affect less than ten genes (Suppl Table 2).

Among the nine chemicals in the PAHs group, BaP (CAS No. 50-32-8) affects the greatest number of genes related to breast cancer development (492 genes), followed by anthracene (CAS No. 120-12-7; 12 genes) (Suppl Table 2). In regard to interactions with lung cancer-associated genes, BaP affects the greatest number of genes (259 genes), followed by benzo(b)fluoranthene (CAS No. 205-99-2; 52 genes), chrysene (CAS No. 218-01-9; 35 genes), dibenz(a, h)anthracene (CAS No. 53-70-3; 34 genes), and benzo(k)fluoranthene (CAS No. 207-08-9; 33 genes) (Suppl Table 2).

### Genes interacted with hydrocarbons mixture that are connected to breast and lung cancers

The data-mining revealed 87 and 44 genes linked to breast and lung cancer, respectively, interacted with most of the investigated hydrocarbons (Suppl Table 3).

The mutual molecular pathways in the development of breast and lung cancer that are linked to these genes are aryl hydrocarbon receptor (AHR) pathway, apoptosis, chemical carcinogenesis, ferroptosis, fluid shear stress and atherosclerosis, lipid and atherosclerosis, miRNA in DNA damage response, Nrf2 pathway, nuclear receptors meta-pathway, and oxidative stress response (Suppl Figs. 1 & 2; Suppl Tables 4 & 5).

Molecular pathways involved in breast cancer but not in lung cancer development are androgen receptor signaling, DNA methylation, estrogen metabolism and signaling, and interleukin-10 (IL-10) anti-inflammatory signaling (Suppl Fig. 1 & Suppl Table 4).

Interleukin-3, 4, 13 and 17 (IL-3, IL-4, IL-13, IL-17) signaling pathways, p53 signaling, oxidative damage response, and SUMOylation are involved in the development of lung cancer (Suppl Fig. 2 & Suppl Table 5) but not breast cancer.

### Gene-gene interaction network affected by the common genes

GeneMANIA Cytoscape predictive plug-in provides information on interaction types between the common genes. The interaction types include: (a) Co-expression—two gene products are linked if their expression levels are similar across conditions in a gene expression study; (b) Genetic interaction—two genes are functionally associated if one gene is affected by alterations that occur to the second gene; (c) Physical Interaction—two genes product are linked if they interact at protein level; (d) Co-localization—genes expressed in the same tissue or proteins found in the same location; (e) Interaction in shared protein domains; and (f) Interaction predicted by the server [18].

Complex networks encompassing the whole set of interactions between the common genes linked to breast and lung cancers are presented in Supplementary Fig. 3. Co-expression (47.33% of interactions) and physical interaction (40.18%) are the dominant interactions among the common genes in breast cancer development, followed by genetic interaction (2.90%), co-localization (2.74%), and interaction in shared protein domains (0.54%) (Table 2). In the case of lung cancer development, co-expression is the dominant interaction between the common genes (46.88%), followed by physical interaction (24.17%), shared protein domain (7.61%), co-localization (7.43%), and genetic interaction (3.04%) (Table 2).

**Table 2:** Type of gene interactions among the common genes linked to breast and lung cancer

Cancer	Gene Interaction Type					
	Co-expression	Genetic Interaction	Physical Interaction	Co-localization	Shared Protein Domain	Predicted
Breast	47.33%	2.90%	40.18%	2.74%	0.54%	4.59%
Lung	46.88%	3.04%	24.17%	7.43%	7.61%	10.48%

In gaining insights on the potential biological pathways that would be affected by exposure to a mixture of hydrocarbons, we focused on 16 genes common in the development of both cancers (Suppl Table 3). Among these genes, 12 are protein-coding genes and 4 are proto-oncogenes.

The protein encoded by the 12 genes are baculoviral inhibitor of apoptosis (IAP) repeat-containing 5 (BIRC5), cyclin D1 (CCNDI), cytochrome P450 1B1 (CYP1B1), DNA methyltransferase 3 alpha (DNMT3A), estrogen receptor 1 (ESR1), glutathione S-transferase pi 1 (GSTP1), heme oxygenase 1 (HMOX1), interleukin 1 β (IL1B), interleukin 6 (IL6), transferrin receptor (TFRC), tumor necrosis factor (TNF), and tumor protein p53 (TP53). The 4 proto-oncogenes are c-Fos (FOS), Jun (JUN), HRas (HRAS) and Kras (KRAS) (Suppl Table 3).

Among the investigated hydrocarbons, 3 are associated with breast cancer development only: the halogenated hydrocarbon, trichloroethylene (TCE) and tetrachloroethylene, and anthracene, a polyaromatic hydrocarbon (Table 3). The chemical-gene interactions involved changes in mRNA and protein expression. All 3 chemicals do not interact with the *HRAS* and *KRAS* genes (Table 3).

**Table 3:** Chemical-gene interaction associated with breast neoplasms

Substance	Trichloroethylene (CRN 79-01-6)			Tetrachloroethylene (CRN 127-18-4)			Anthracene (CRN 120-12-7)		
Interaction	mRNA Expr	Protein		mRNA Expr	Protein		mRNA Expr	Protein	
		Expr	Activity		Expr	Activity		Expr	Activity
*BIRC5*	↑	↑		↑					
*CCND1*	↑↓	↑						↑	
*CYP1B1*	↑↓						↑		
*DNMT3A*	↓								
*ESR1*	↑							↑	
*FOS*	↑				↓				
*GSTP1*	↑								
*HMOX1*	↑	↓							
*HRAS*									
*IL1B*	↑	↑							
*IL6*	↑↓	↑↓							
*JUN*	↑↓	↑			↓				
*KRAS*									
*TFRC*	↓								
*TNF*	↑↓	↑↓			↑				
*TP53*	↓	↑		↑					

Expr: Expression; ↑ - increase; ↓ - decrease; ↑↓ - can both increase and decrease. The references link can be found in Supplementary Table 2

TCE interacted with the other 14 genes: it increased the expression of mRNA and/or protein of BIRC5 [31, 32], CCND1 [33], CYP1B1 [34], ESR1 [35], FOS [35], GSTP1 [35], HMOX1 [31, 35, 36], IL1B [37], IL6 [35], JUN [31, 35, 37], TNF [38], and TP53 [39]. It also decreased mRNA expression of CCND1 [40], CYP1B1 [36], DNMT3A [31, 41], IL6 [42], JUN [43], TFRC [35], TNF [44], TP53 [34], and decreased HMOX1, IL6 and TNF protein expression [38, 45] (Table 3). The potential biological pathways affected by TCE are AHR pathway, apoptosis, chemical carcinogenesis, DNA methylation, estrogen metabolism and signaling, ferroptosis, fluid shear stress & atherosclerosis, IL-10 anti-inflammatory signaling, lipid and atherosclerosis, miRNA in DNA damage, Nrf2 pathway, and oxidative stress response (Fig. 2).

Tetrachloroethylene interacted with only 5 genes (*BIRC5, FOS, JUN*, TNF, and TP53). It increased the expression of BIRC5 and TP53 mRNAs and TNF protein, as well as decreased the expression of FOS and JUN proteins (Table 3). The potential biological pathways affected by tetrachloroethylene are AHR pathway, apoptosis, estrogen signaling, ferroptosis, fluid shear stress & atherosclerosis, lipid and atherosclerosis, miRNA in DNA damage, nuclear receptors meta-pathway, and oxidative stress response (Fig. 2). Tetrachloroethylene ability to increase the expression of *BIRC5* mRNA (Table 3) suggests deregulation of apoptosis as a potential mechanism affected by tetrachloroethylene in the development of breast cancer. This is because BIRC5—a member of the inhibitor of apoptosis (IAP) family—inhibits caspase activation, which leads to deregulation of apoptosis and increase cellular proliferation [46].

Anthracene interacted with only 3 genes (*CCND1, CYP1B1*, and *ESR1*). It increased the expression of CYP1B1 mRNA, as well as CCND1 and ESR1 protein (Table 3). The potential biological pathways affected by anthracene are AHR pathway, chemical carcinogenesis, estrogen metabolism and signaling, estrogen-dependent nuclear events, miRNA in DNA damage, and nuclear receptors meta-pathway (Fig. 2).

In the case of lung cancer, the interactions of carbon tetrachloride, vinyl chloride,dibenz(a, h)anthracene, benzo(b)fluoranthene, benzo(k)fluoranthene, and chrysene with the 16 genes, involved up- and down-regulation at mRNA and protein levels, gene polymorphism, and gene mutagenesis (Table 4).

**Table 4:** Chemical-gene interaction associated with lung neoplasms

Substance	Carbon tetrachloride (CRN 56-23-5)			Vinyl chloride (CRN 75-01-4)			Dibenz(a, h)anthracene (CRN 53-70-3)			Benzo(b)fluoranthene (CRN 205-99-2)			Benzo(k)fluoranthene (CRN 207-08-9)			Chrysene (CRN 218-01-9)		
Interaction	mRNA Expr	Protein		mRNA Expr	Protein		mRNA Expr	Protein		mRNA Expr	Protein		mRNA Expr	Protein		mRNA Expr	Protein	
		Expr	Activity		Expr	Activity		Expr	Activity		Expr	Activity		Expr	Activity		Expr	Activity
*BIRC5*	↑	↑					↑			↑	↑		↑			↑		
*CCND1*	↑↓	↑					↑			↑			↑			↑		
*CYP1B1*	↑						↑	↑		↑			↑			↑	↑	
*DNMT3A*			↑							↑								
*ESR1*	↑↓										↑	↑		↓		↑		
*FOS*	↑↓	↑	↑										↑			↑		
*GSTP1* ^*^	↑	↑	↓				↓			↑			↑			↑		
*HMOX1*	↑↓	↑↓	↑	↓	↓					↑			↑↓					
*HRAS*	↑																	
*IL1B*	↑	↑	↑	↓						↑			↑					
*IL6*	↑	↑	↑	↓						↑		↑				↑		
*JUN*	↑	↑	↑										↑			↑		
*KRAS* ^**^	↑↓																	
*TFRC*		↓											↑					
*TNF*	↑	↑	↑	↑		↑										↑		
*TP53* ^***^	↑↓			↓	↑						↑	↑↓			↑↓			

Expr: Expression; ↑ - increase; ↓ - decrease; ↑↓ - can both increase and decrease; ^*^GSTP1 polymorphism ↑ susceptibility to vinyl chloride (117); ^**^Vinyl chloride (44–47) and benzo(b)fluoranthene (50–51) ↑ mutagenesis of *KRAS* gene; ^***^Vinyl chloride ↑ mutagenesis *TP53* gene (45–46). TP53 protein increased susceptibility to dibenz(a, h)anthracene (62). The references link can be found in Supplementary Table 2

Carbon tetrachloride affected the up- and or down-regulation of all 16 genes at mRNA and or protein levels (Table 4). It increased the activity of DNMT3A, FOS, HMOX1, IL1B, IL6, JUN, and TNF. This implicates potential involvement of biological pathways associated with AHR pathway, apoptosis, chemical carcinogenesis, ferroptosis, fluid shear stress & atherosclerosis, oxidative stress response, and SUMOylation (Fig. 3).

Vinyl chloride interacted with only 6 genes. In addition to down regulating HMOX1, IL1B, IL6, and TP53 at mRNA level, as well as decreasing HMOX1 protein expression, it increased mutagenesis of both *KRAS* [47–50] and *TP53* [47–49] genes (Table 4). When *KRAS* gene is mutated, it becomes an oncogene that can transform normal cells into cancer cells [51], whilst TP53 mutations resulted in uncontrolled cell growth leading to cancer development [52]. Thus, the potential mechanism by which vinyl chloride contributes to the development of lung cancer is associated with disruption of normal cellular processes and promotion of tumorigenesis. In the case of TNF, vinyl chloride increased the mRNA and protein activity (Table 4), indicating potential impact in tumor microenvironment.

Among the 4 PAHs associated with lung cancer, benzo(b)fluoranthene and benzo(k)fluoranthene interacted with 11 of the 16 genes, whilst chrysene and dibenz(a, h)anthracene interacted with 5 and 4 genes, respectively (Table 4). The upregulation of DNMT3A mRNA was increased by benzo(b)fluoranthene but not by the other 3 PAHs (Table 4). Similarly, the regulations of KRAS mRNA and protein were unaffected by all 4 PAHs, except for benzo(b)fluoranthene increased the mutagenesis of *KRAS* gene [53, 54]. It also increased TP53 protein expression and affected its activity [55] (Table 4). This suggests that benzo(b)fluoranthene and vinyl chloride affected similar biological pathways in the development of lung cancer. A comprehensive overview of the interaction between the 16 genes and PAHs that are linked to lung cancer is shown in Fig. 3.

In the development of both cancers, TCDD and BaP interacted with all 16 genes by affecting the respective mRNA and protein expression and or protein activity (Table 5). BaP can also affect the methylation of *BIRC5* 3’UTR, *GSTP1* promoter, *HRAS* and *IL1B* 5’UTR, and phosphorylation of TP53 protein [56–58]. The alkenes, isoprene and 1,3-butadiene increased the mutagenesis of both *HRAS* and *KRAS* genes [59], whilst BaP increased the mutagenesis of *KRAS* gene [53, 54, 60, 61] (Table 5). Isoprene also increased the expression of CCND1 protein (Table 5). This indicates similar mechanism of actions by which isoprene, 1,3-butadiene and BaP contribute to the development of both breast and lung cancers.

**Table 5:** Chemical-gene interaction associated with breast and lung neoplasms^ŧ^

Substance	2-Methyl-1,3-butadiene (CAS No. 78-79-5)			1,3-Butadiene (CAS No. 106-99-0)			Dichloromethane (CAS No. 75-09-2)			Tetrachlorodibenzodioxin (CAS No. 1746-01-6)			Benzo(a)pyrene (CAS No. 50-32-8)		
Interaction	mRNA Expr	Protein		mRNA Expr	Protein		mRNA Expr	Protein		mRNA Expr	Protein		mRNA Expr	Protein	
		Expr	Activity		Expr	Activity		Expr	Activity		Expr	Activity		Expr	Activity
*BIRC5* ^*^				↑						↑↓	↑		↑↓	↓	
*CCND1*		↑								↑↓	↑↓		↑↓	↑↓	
*CYP1B1*							↓			↑	↑	↑	↑↓	↑	↑
*DNMT3A*				↓						↑↓		↑	↑↓	↑	
*ESR1*							↓			↑↓	↑↓	↑↓	↑↓	↓	↑
*FOS*				↓			↓			↑↓	↑↓	↑	↑↓	↑	
*GSTP1* ^**^				↑						↑↓		↑	↑↓	↑	↑
*HMOX1*				↑			↑			↑↓	↑↓		↑	↑↓	
*HRAS* ^***^										↑↓	↑		↑↓	↑	
*IL1B* ^****^				↓						↑	↑		↑	↑	↑
*IL6*								↑		↑↓	↑↓	↑	↑	↑	↑
*JUN*							↑			↑↓	↑↓	↑	↑↓	↑	↑
*KRAS* ^#^										↑↓	↑		↑		
*TFRC*				↑						↑↓	↑		↑↓		
*TNF*								↑		↑	↑↓	↑	↑	↑	↑
*TP53* ^###^										↑↓	↓	↓	↑↓	↑↓	↑

Expr: Expression; ↑ - increase; ↓ - decrease; ↑↓ - can both increase and decrease; ^ŧ^1,2-Dichloropropane (CAS No. 78-87-5) interacted with only one common gene, *TNF*. It ↑ the expression of TNF mRNA; ^*^BaP ↑ methylation of BIRC5 3’UTR (53); ^**^BaP ↓ methylation of *GSTP1* promoter (53); ^***^2-Methyl-1,3-butadiene (isoprene) and 1,3-butadiene ↑ mutagenesis of *HRAS* gene (1). BaP ↓ methylation of HRAS 5’UTR (53); ^****^BaP ↓ methylation of IL1B 5’UTR (53). ^#^Isoprene, 1,3-butadiene and BaP ↑ mutagenesis of *KRAS* gene (50–51,57–59); ^##^BaP ↓ methylation of MIR222 gene promoter (53); ^###^BaP ↑ phosphorylation of TP53 protein (54–56). The references link can be found in Supplementary Table 2.

## Discussion

In utilizing the in silico toxicogenomic data-mining approach—to explore molecular mechanisms by which exposure to hydrocarbons mixture affects cancer development—we identified 16 genes common in the development of breast and lung cancers that interact with most of the investigated hydrocarbons. Proteins encoded by these genes: *BIRC5*, *CCND1*, *TNF*, and the proto-oncogenes *FOS*, *JUN*, *HRAS*, and *KRAS*, have all been implicated in cell cycle regulation directly or indirectly. The other 9 genes, *CY1B1*, *DNMT3A, ESR1, GSTP1, HMOX1*, *IL1B, IL6, TFRC, and TP53* encode proteins involved in xenobiotic metabolism, gene regulation, oxidative damage response, inflammatory response, iron homeostasis, regulation of cell signaling pathways, and DNA damage response.

It is noted that these common genes may interact with carcinogens other than the investigated hydrocarbons. For example, acetaldehyde (CAS No. 75-07-0), asbestos (CAS No. 1332-21-4), bis(2-ethyl hexyl) phthalate (CAS No. 117-81-7), cadmium (CAS No. 7440-43-9), and chromium (CAS No. 18540-29-9) interacted with most of the 16 common genes, except *BIRC5, DNMT3A, HRAS* and *KRAS* (Suppl Table 6). The latter 4 genes interacted with carbon tetrachloride, 1,3-butadiene, TCDD, and BaP in the development of both breast and lung cancers.

The chemical-gene interactions profile suggests complex crosstalk involving DNA damage, transcriptional and post-transcriptional regulations, as well as translational and post-translational regulations, that affect various biological pathways common to cancer development. These biological pathways include gene mutation, cell cycle progression, oxidative stress and damage responses, inflammatory responses, and DNA damage responses.

In our mapping of biological pathways for breast cancer, DNMT3A, an enzyme responsible for de novo DNA methylation, is shown to be involved in miRNA expression (Suppl Fig. 1). The crosstalk between DNA methylation and miRNA expression can drive the pathogenesis of a disease. For instance, miRNAs can influence DNA methylation patterns by targeting transcripts of proteins responsible for DNA methylation, such as DNMT3A. Conversely, the methylation of miRNA promoter regions can inhibit their transcription, affecting their ability to regulate gene expression. Such crosstalk has been shown to drive the hormone-dependent phenotype of breast cancer [62].

In the case of lung cancer, the function of DNMT3A may be modified by SUMOylation a process of attaching and detaching small proteins called Small Ubiquitin-like Modifier (SUMO) to and from target proteins. This may lead to changes in the methylation of genes involved in cell growth and division, potentially contributing to uncontrolled cell proliferation. SUMOylation of other target proteins has been shown to enhance lung cancer metastasis [63].

The nature of chemical interaction in a mixture of hydrocarbons, such as additive, synergistic, potentiation, and antagonism cannot be discerned from this study due to inherent limitations of the study approach. However, the differences in chemical-gene interactions observed among the hydrocarbons provide insights into potential impact of exposure to hydrocarbons mixture.

For example, TCE—a halogenated hydrocarbon associated with increased risk of breast cancer in male and female workers [64, 65]—may potentiate the risk of lung cancer from exposure to dibenz(a, h)anthracene and the risk of both breast and lung cancer from exposure to BaP. The potential mechanism for such potentiation is increased DNA damage through DNA adduct formation and increased cellular proliferation through deregulation of apoptosis. TCE upregulates TP53 protein expression [39], as well as BIRC5 mRNA and protein expression [31, 32]. Elevated cellular TP53 protein has been shown to increase bioactivation of PAHs, such as dibenz(a, h)anthracene and BaP, by the enzyme cytochrome P450 1A1 (CYP1A1), which resulted in the elevation of DNA adduct levels [66]. BIRC5, on the other hand, inhibits caspase activation, which leads to deregulation of apoptosis and increase cellular proliferation [39].

In the case of vinyl chloride co-exposed with dibenz(a, h)anthracene and BaP, the DNA adduct formation via p53-dependent CYP1A1 bioactivation of the two PAHs, may be reduced as vinyl chloride is known to increase mutagenesis of the *TP53* gene [48, 49].

### Chemical carcinogenesis

Chemical carcinogenesis that pivots on the AHR pathway appears to be the bridge linking the development and progression of breast and lung cancers. AHR plays a "double-edged sword" that promotes or suppresses tumorigenesis, depending on cell and tissue context and mode of AHR activation. In breast cancer, AHR shapes the tumor microenvironment and modifies immune tolerance [67], whilst in lung cancer, AHR is involved in the regulation of cell proliferation, angiogenesis, inflammation, and apoptosis [68].

AHR is a multi-functional transcription factor activated by a variety of ligands, such as BaP, benz(a)anthracene, TCDD, and metabolites of tryptophan, heme and arachidonic acid, indigoids, and equilenin (reviewed in [69]). These ligands can be agonist, antagonist or selective AHR modulators [70]. Upon ligand binding, the cytosolic AHR-ligand complex is translocated into the nucleus where it heterodimerizes with the aryl hydrocarbon nuclear transporter (ARNT) before binding to the xenobiotic/dioxin response elements (XREs/DREs) in the promoter of target genes and triggers their expression [71]. These genes are involved in many physiological functions, such as xenobiotic metabolism [71], immune response [72], cell cycle and proliferation [73, 74], lipid metabolism [75, 76], tumor promotion [77, 78], and negative regulation of AHR pathway [76]. Perturbations of these physiological functions have been shown to be associated with cancer development and progression, which suggests a complex role of AHR in chemical carcinogenesis.

In xenobiotic metabolism, AHR activates transcriptional up-regulation of the cytochrome P450 1A1 (*CYP1A1)* and *CYP1B1* genes. Most of the investigated hydrocarbons are linked to these two genes. Some are substrates for both enzymes. For example, the first step of BaP hydroxylation to BaP-7,8-epoxide, and the final epoxidation step to form BaP-7,8-dihydrodiol-9,10-epoxide (BPDE) are catalyzed by CYP1A1 and CYP1B1 enzymes in the lung and breast tissues [79–81], BPDE is a highly genotoxic metabolite that binds to deoxyguanosine at position N-2 to form DNA adducts [82]. Cigarette smoke was reported to induce *CYP1A1* and *CYP1B1* expressions in lung tissue of smokers and of lung cancer patients (both smokers and non-smokers) [83–85], which correlates with increased levels of BPDE and DNA adducts [86–90]. Bulky BaP-like DNA adducts were also detected in breast cancer patients [91, 92]. PAHs reactive metabolites are known to cause point mutations in RAS proto-oncogenes, such as codon 13 and codon 61 of the HRAS gene (reviewed in [93]). These observations suggest that the AHR/CYP450-dependent DNA adducts formation is a likely pathway to be affected by exposure to hydrocarbons mixture in the development of breast and lung cancers.

Several mechanisms by which AHR modulates the cell cycle have been proposed to account for the pro-/anti-proliferative action of AHR agonists observed with tumor cells in vitro [67, 68, 78]. One of the proposed ligand-activated mechanisms involved transcriptional upregulation of the *CDKN1B* gene by agonist-activated AHR binding to the gene’s promoter region [94, 95]. However, this mechanism has not been demonstrated in the development and progression of either breast or lung cancer. Being an inhibitor of cyclin-dependent kinase activity, increased CDKN1B activity limits phosphorylation of retinoblastoma protein (Rb), resulting in restriction of E2F-dependent gene expression and progression through the cell cycle [70]. In the absence of ligand, AHR complexed with cyclin D and the cyclin-dependent kinases CDK4/6 to promote cell cycle progression in human breast cancer cells [96]. TCDD, the atypical AHR agonist, dissociates the AHR/cyclinD/CDK complex to induce cell cycle arrest [96]. This contradictory role of AHR may reflect the impact of exposure to hydrocarbon mixtures on cell proliferation, as most of the investigated hydrocarbons are AHR agonists with different affinity to the receptor [70]. Similar contradictory effects of AHR on cell cycle progression were also observed in human lung cancer cells [97]. The impact of exposure to mixtures of 2-methyl-1,3-butadiene, carbon tetrachloride, TCDD, and BaP on this pathway may contribute to the development of breast and or lung cancer as these hydrocarbons interacted with *CDKN1B* gene (Suppl Table 2).  

The mechanisms by which AHR shapes the tumor microenvironment are unclear, but it has been proposed that systemic and tumor-localized generation of endogenous AHR ligands heightened AHR expression/activity, which may establish a pro-inflammatory yet immune-suppressive tumor micro-environment. This favors tumor survival and escapes from immune surveillance, which results in tumor progression [70]. Indeed, AHR overexpression that is correlated with elevated expression of inflammatory markers, including interleukin-8 (IL-8), has been observed in human breast tumors [98]. *IL-8* has been identified as a critical gene that mediates breast cancer invasion and metastasis to the lungs [99]. The involvement of *IL1B*—one of the 16 common genes identified in this study—in this mechanism has not been elucidated in both breast and lung cancer development and progression. Our mapping, however, showed *IL1B* is involved in modulating the IL-17 signaling pathway, lipid and atherosclerosis pathway, and fluid shear stress and atherosclerosis pathway in both breast and lung cancer development. It is plausible that the impact of PAHs mixture on these pathways may involve AHR activation, as fluid shear stress in endothelial cells has been shown to modulate CYP1A-dependent AHR activation [100, 101], but the mechanism of activation remains unclear [67, 68].

### Adaptive responses to cellular damage

AHR activation has also been shown to be associated with the oxidative stress response pathway. For example, exposure of estrogen receptor (ER) positive breast cancer cells to low doses of PAHs mixture activated AHR and overexpressed CYP1 isoforms, which correlated with increased expression of antiapoptotic and antioxidant proteins [102].

Besides catalyzing the biotransformation of PAHs to DNA damaging reactive metabolites, CYP1A1 and CYP1B1 catalyze the oxidation of estradiol (E2) to 2-hydroxyestradiol and 4-hydroxyestradiol, which subsequently undergo one-electron oxidation to produce unstable semiquinones (SQs) intermediates [103], potential mutagens that can damage DNA [104, 105]. Additionally, redox cycling can occur, where the SQs can pass their unpaired electron to molecular oxygen, forming a superoxide anion and restoring the catechol. Superoxide anion can then be metabolized to other reactive oxygen species (ROS), including hydrogen peroxide (H_2_O_2_) [103, 105]. Another contributor of ROS in breast cancer cells is the expression of *CYP2E1*, which increased significantly in breast tumors and adjacent tissues [106]. CYP2E1 also regulates autophagy, stimulates stress in the endoplasmic reticulum, and suppresses the metastatic potential of breast cancer cells [107], indicative of the protective role of CYP2E1.

Excessive ROS can cause DNA damage, as well as lipid and protein oxidation, which triggers an oxidative stress response that involves activation of the NRF2-KEAP1 signaling pathway. This pathway modulates the expression of genes encoding antioxidant proteins, such as superoxide dismutase and HMOX1. The latter is involved in the maintenance of cellular homeostasis by catalyzing the oxidation of heme to carbon monoxide, biliverdin, and ferrous iron. These biologically active compounds participate in cellular protection by reducing oxidative injury, attenuating the inflammatory response, inhibiting cell apoptosis, and regulating cell proliferation [108]. In mouse, HMOX1 activity increased tumor growth and angiogenic potential, as well as decreased apoptosis in lung cancer progression [109], whilst in rat and human breast cancer cells, HMOX1 activity inhibits proliferation [110].

Notably, the NRF2-KEAP1 signaling pathway is linked to ferroptosis, one of the common pathways in the development of breast and lung cancer mapped out in this study.

### Ferroptosis

Ferroptosis is a non-apoptotic programmed cell death, which has gained traction as a new target for treating tumors [111]. It is regulated by a complex signaling pathway that is dependent on lipid peroxidation and iron accumulation [111, 112]. Evidence that supports the potential physiological roles of ferroptosis in tumorigenesis resides in the way it is induced in cancer cells. This includes activation of the RAS–RAF–MEK–ERK pathway and induction in cancer cells with mutant RAS, as well as dependency on iron, which is known to be important for cancer cell proliferation (reviewed in [112]). Induction of ferroptosis has been shown to suppress tumor growth, but ferroptotic damage favors tumor growth by triggering inflammation-associated immunosuppression in the tumor microenvironment [112]. Therefore, the three key features of ferroptosis: iron accumulation, increased lipid peroxidation and inability to efficiently reduce lipid peroxidases, must be well regulated to strike the delicate balance of survival and damage in tumorigenesis.

Little is known about ferroptosis role in breast and lung cancer progression, and the impact of exposure to hydrocarbons mixture on such association. However, several studies have found important correlations between mutations in tumor suppressor gene and proto-oncogene, *TP53* and *RAS*, and in genes encoding proteins involved in stress response pathways. One of these pathways is the NRF2 signaling pathway [112], which is one of the common molecular pathways identified in this study.

Depending on the pathological condition, the transcription factor NRF2 serves as either an anti- or pro-ferroptotic activator. Under oxidative stress conditions, NRF2 complexed with its chaperon protein to bind to the ARE (Antioxidant Response Element) on the promoter region of its target genes with anti- or pro-ferroptotic functions. An example of iron-related NRF2 target gene that promotes ferroptotic cascade is HMOX1, which catalyzes the cleavage of heme to form biliverdin, carbon monoxide, and ferrous iron (Fe^2+)^ [113]. Chemical-induced ferroptotic cell death driven by increased HMOX1 expression was observed in HT-1080, neuroblastoma and glioblastoma cell lines [114–116]. An example of NRF2 acting as anti-ferroptotic activator is in its regulating the expression of enzymes responsible for glutathione synthesis, as well as preventing lipid peroxidation and reducing oxidized CoQ10, a key membrane antioxidant (GPX4 and FSP1) [113]. Notably, GSTP1 has been shown to be involved in tumor development through the ferroptosis pathway [117] and was suggested to be a novel negative regulator of ferroptosis that may play an important role in lung cancer radiotherapy by inhibiting ferroptosis [118]. Crosstalk mechanisms between the RAS–RAF–MEK–ERK pathway and the NRF2 signaling pathway, with the involvement of GSTP1 in ferroptosis during tumorigenesis in breast and lung cells, and impact of exposure to halogenated and polyaromatic hydrocarbons on the crosstalk mechanisms have yet to be explored.

The role of AHR and NRF2 in regulating ferroptosis in breast and lung cancer cells is unclear, but AHR has been shown to promote the development of non-small cell lung cancer (NSCLC) by inducing the expression of *SLC7A11*, a key regulator of ferroptosis [119].

In sum, as most hydrocarbons are AHR ligands, the impact of inhalation exposure to hydrocarbons mixture on these physiological functions is complex, given the distinct classes of AHR ligands: agonist, antagonist and selective AHR modulators [70]. However, this study revealed an important role of AHR in being the bridge linking the development and progression of breast and lung cancers as it is involved (directly and or indirectly) in the regulation of biological pathways mapped out in this study. Notably, the mechanism by which IL1B regulates *IL-8*—a critical gene that mediates breast cancer invasion and metastasis to the lungs [99]—and the role of AHR in such mechanism, is worth pursuing.

## Conclusion

Within the inherent limitations of in silico toxicogenomics associated tools, we were able to elucidate the molecular pathways of breast and lung cancer development potentially affected by exposure to hydrocarbons mixture. In silicon data-mining depends on the online sources and the quality of the interactions present in them. Complex molecular pathways were obtained by drawing statistical associations between chemical-gene-disease relationships. Therefore, dose-response relationship, interaction profile of hydrocarbons mixture, route and duration of exposure to the investigated hydrocarbons mixture, along with individual sensitivity of exposed subjects, cannot be drawn from this study. In conclusion,  our findings should be regarded as insights into future in vivo and in vitr laboratory investigations that focus on inhalation exposure to the hydrocarbons mixture.

### Electronic supplementary material

Below is the link to the electronic supplementary material.

Supplementary Material 1

Supplementary Material 2

Supplementary Material 3

Supplementary Material 4

Supplementary Material 5

Supplementary Material 6

Supplementary Material 7

Supplementary Material 8

Supplementary Material 9

## Data Availability

All data generated or analyzed during this study are included in this published article. Additional supplementary data are available from the corresponding author upon request.

## Abbreviations

A2M: Alpha-2-macroglobulin
ABCA8: ATP binding cassette subfamily A member 8
ABCB1: ATP binding cassette subfamily B member 1
ABCB1B: ATP-binding cassette, sub-family B
ABCC1: ATP binding cassette subfamily C member 1
ABCG2: ATP binding cassette subfamily G member 2
ABL1: Abl tyrosine kinase proto-oncogene 1
ACACB: Acetyl-CoA carboxylase beta
ACE: Angiotensin I converting enzyme
ACHE: Acetylcholinesterase
ACSM1: Acyl-CoA synthetase medium chain family member 1
ACTA2: Actin alpha 2
ACTB: Actinβ
ACVR1: Activin A receptor type 1
ADA: Adenosine deaminase
ADAR: Adenosine deaminase RNA specific
ADAM10: ADAM metallopeptidase domain 10
ADAM28: ADAM metallopeptidase domain 28
ADAM33: ADAM metallopeptidase domain 33
ADAMTS1: ADAM metallopeptidase with thrombospondin type 1 motif 1
AFP: Alpha fetoprotein
AGR2: Anterior gradient 2
AHR: Aryl hydrocarbon receptor
AKAP12: A-kinase anchoring protein 12
AKT1: Akt kinase
AKT2: AKT serine/threonine kinase 2
ALDOA: Aldolase, fructose-bisphosphate A
ALK: ALK receptor tyrosine kinase
ALKBH8: AlkB homolog 8
ALX4: ALX homeobox 4
ANGPTL4: Angiopoietin like 4
ANK3: Ankyrin 3
ANKRD18A: Ankyrin repeat domain 18 A
ANKRD20A2P: Ankyrin repeat domain 20 family member A2
ANKRD34A: Ankyrin repeat domain 34 A
ANXA2: Annexin A2
AOC4P: Amine oxidase copper containing 4,pseudogene
APC: APC regulator of WNT signaling pathway
APC2: APC regulator of WNT signaling pathway 2
APOA1: Apolipoprotein A1
APOBEC3A: Apolipoprotein B mRNA editing enzyme catalytic subunit 3 A
APOBEC3B: Apolipoprotein B mRNA editing enzyme catalytic subunit 3B
APOC3: Apolipoprotein C3
APOE: Apolipoprotein E
APRT: Adenine phosphoribosyltransferase
AR: Androgen receptor
ARAF: A-Raf proto-oncogene
AREG: Amphiregulin
ARF1: ADP ribosylation factor 1
ARHGDIA: Rho GDP dissociation inhibitorα
ARHGEF5: Rho guanine nucleotide exchange factor 5
ARID1A: AT-rich interaction domain 1 A
ARRDC3: Arrestin domain containing 3
ARTN: Artemin
AS3MT: Arsenite methyltransferase
ATG10: Autophagy related 10
ATG101: Autophagy related 101
ATM: ATM serine/threonine kinase
ATOX1: Antioxidant 1 copper chaperone
ATP6AP1L: ATPase H + transporting accessory protein 1 like (pseudogene)
ATP7B: ATPase copper transportingβ
ATSDR: Agency for Toxic Substances and Disease Registry
AURKA: Aurora kinase A
AVPI1: Arginine vasopressin induced 1
AZGP1: α-2-glycoprotein 1,zinc-binding
B4GAT1: Beta-1,4-glucuronyltransferase 1
BAG1: BAG cochaperone 1
BAP1: BRCA1 associated protein 1
BARD1: BRCA1 associated RING domain 1
BAX: CL2 associated X
BCAR3: BCAR3 adaptor protein
BCHE: Butyrylcholinesterase
BCL2: BCL2 apoptosis regulator
BCL2A1: BCL2 related protein A1
BCL2L1: BCL2 like 1
BECN1: Beclin 1
BGN: Biglycan
BHLHE41: Basic helix-loop-helix family member e41
BIRC2: Baculoviral IAP repeat containing 2
BIRC5: Baculoviral IAP repeat containing 5
BMP2: Bone morphogenetic protein 2
BMP4: Bone morphogenetic protein 4
BMPR2: Bone morphogenetic protein receptor type 2
BRAF: B-Raf proto-oncogene, serine/threonine kinase
BRCA1: BRCA1 DNA repair associated
BRCA2: BRCA2 DNA repair associated
BRF1: BRF1 RNA polymerase III transcription initiation factor subunit
BRIP1: BRCA1 interacting helicase 1
BTN3A2: Butyrophilin subfamily 3 member A2
C1QBP: Complement C1q binding protein
CA12: Carbonic anhydrase 12
CADM1: Cell adhesion molecule 1
CALEPA: California Environmental Protection Agency
CALML3: Calmodulin like 3
CAS: Chemical Abstract Service
CASP7: Caspase 7
CASP8: Caspase 8
CAT: Catalase
CAV1: Caveolin 1
CBR2: Carbonyl reductase 2
CCL18: C-C motif chemokine ligand 18
CCL20: C-C motif chemokine ligand 20
CCN1: Cellular communication network factor 1
CCN2: Cellular communication network factor 2
CCND1: Cyclin D1
CCNE1: Cyclin E1
CCNG1: Cyclin G1
CCNH: Cyclin H
CCT5: Chaperonin containing TCP1 subunit 5
CD109: CD109 molecule
CD274: CD274 molecule
CD40: CD40 molecule
CD74: CD74 molecule
CDA: Cytidine deaminase
CDH1: Cadherin 1
CDH13: Cadherin 13
CDH2: Cadherin 2
CDH5: Cadherin 5
CDKN1A: Cyclin dependent kinase inhibitor 1 A
CDKN1B: Cyclin dependent kinase inhibitor 1B
CDKN1C: Cyclin dependent kinase inhibitor 1 C
CDKN2A: Cyclin dependent kinase inhibitor 2 A
CEACAM1: CEA cell adhesion molecule 1
CENPF: Centromere protein F
CES1: Carboxylesterase 1
CES1F: Carboxylesterase 1 F
CFL1: Cofilin 1
CHD4: Chromodomain helicase
DNA: Binding protein 4
CHEK1: Checkpoint kinase 1
CHEK2: Checkpoint kinase 2
CHRNA2: Cholinergic receptor nicotinic α 2 subunits
CHRNA3: Cholinergic receptor nicotinic α 3 subunits
CHRNA5: Cholinergic receptor nicotinic α 5 subunits
CHRNA7: Cholinergic receptor nicotinic α 7 subunits
CHRNB4: Cholinergic receptor nicotinic β 4 subunits
CHST15: Carbohydrate sulfotransferase 15
CLCA2: Chloride channel accessory 2
CLDN1: Claudin 1
CLDN4: Claudin 4
CLIC1: Chloride intracellular channel 1
CLPTM1L: Cleft lip and palate associated transmembrane protein 1
CLTB: Clathrin light chain B
CLUL1: Clusterin like 1
CNR2: Cannabinoid receptor 2
COL6A1: Collagen type VI α 1 chain
COL7A1: Collagen type VII α 1 chain
COMT: Catechol-O-methyltransferase
COTL1: Coactosin like F-actin binding protein 1
COX17: Cytochrome c oxidase copper chaperone COX17
CPE: Carboxypeptidase E
CPT1A: Carnitine palmitoyltransferase 1 A
CRHR1: Corticotropin releasing hormone receptor 1
CRP: C-reactive protein
CSF1: Colony stimulating factor 1
CSF1R: Colony stimulating factor 1 receptor
CSF2: Colony stimulating factor 2
CSF3: Colony stimulating factor 3
CST6: Cystatin E/M
CTD: Comparative Toxicogenomic Database
CTNNB1: Catenin beta 1
CTU1: Cytosolic thiouridylase subunit 1
CTU2: Cytosolic thiouridylase subunit 2
CUL5: Cullin 5
CWH43: Cell wall biogenesis 43 C-terminal homolog
CXCL1: C-X-C motif chemokine ligand 1
CXCL12: C-X-C motif chemokine ligand 12
CXCL14: C-X-C motif chemokine ligand 14
CXCL2: C-X-C motif chemokine ligand 2
CXCL3: C-X-C motif chemokine ligand 3
CXCL8: C-X-C motif chemokine ligand 8
CXCL9: C-X-C motif chemokine ligand 9
CXCR4: C-X-C motif chemokine receptor 4
CYP17A1: Cytochrome P450 family 17 subfamily A member 1
CYP19A1: Cytochrome P450 family 19 subfamily A member 1
CYP1A1: Cytochrome P450 family 1 subfamily A member 1
CYP1A2: Cytochrome P450 family 1 subfamily A member 2
CYP1B1: Cytochrome P450 family 1 subfamily B member 1
CYP24A1: Cytochrome P450 family 24 subfamily A member 1
CYP2A6: Cytochrome P450 family 2 subfamily A member 6
CYP2B1: Cytochrome P450 family 2 subfamily B member 1
CYP2D6: Cytochrome P450 family 2 subfamily D member 6
CYP2E1: Cytochrome P450 family 2 subfamily E member 1
CYP3A4: Cytochrome P450 family 3 subfamily A member 4
DAB2IP: DAB2 interacting protein
DAP3: Death associated protein 3
DAPK1: Death associated protein kinase 1
DDIT3: DNA damage inducible transcript 3
DDR1: Discoidin domain receptor tyrosine kinase 1
DEK: DEK proto-oncogene
DEPP1: DEPP autophagy regulator 1
DES: Desmin
DHFR: Dihydrofolate reductase
DIO3: iodothyronine deiodinase 3
DKK1: Dickkopf WNT signaling pathway inhibitor 1
DLL1: Delta like canonical Notch ligand 1
DLL4: Delta like canonical Notch ligand 4
DLL4: Delta like canonical Notch ligand 4
DNAI7: Dynein axonemal intermediate chain 7
DNASE1L3: Deoxyribonuclease 1 like 3
DNMT1: DNA methyltransferase 1
DNMT3A: DNA methyltransferase 3α
DNMT3B: DNA methyltransferase 3β
DOK1: Docking protein 1
DOK2: Docking protein 2
DOK3: Docking protein 3
DPYD: Dihydropyrimidine dehydrogenase
DSC3: Desmocollin 3
DTX3: Deltex E3 ubiquitin ligase 3
DYNC2H1: Dynein cytoplasmic 2 heavy chain 1
E2F1: E2F transcription factor 1
EAF2: ELL associated factor 2
ECHA: European Chemicals Agency
EDNRB: Endothelin receptor type B
EEF1B2: Eukaryotic translation elongation factor 1 beta 2
EEF2: Eukaryotic translation elongation factor 2
EFEMP1: EGF containing fibulin extracellular matrix protein 1
EFNA1: Ephrin A1
EFNB2: Ephrin B2
EGF: Epidermal growth factor
EGFR: Epidermal growth factor receptor
EGR1: Early growth response 1
EHMT2: Euchromatic histone lysine methyltransferase 2
EIF2S2: Eukaryotic translation initiation factor 2 subunitβ
EIF6: Eukaryotic translation initiation factor 6
ELK3: ETS transcription factor ELK3
ELP1: Elongator acetyltransferase complex subunit 1
ELP3: Elongator acetyltransferase complex subunit 3
EMSY: EMSY transcriptional repressor, BRCA2 interacting
EMX2: Empty spiracles homeobox 2
ENO1: Enolase 1
EP300: E1A binding protein p300
EPA/RPF: USEPA relative potency factor applied
EPB41L3: Erythrocyte membrane protein band 4.1 like 3
EPHB4: EPH receptor B4
EPHX1: Epoxide hydrolase 1
EPOR: Erythropoietin receptor
ERBB2: Erb-b2 receptor tyrosine kinase 2
ERBB3: Erb-b2 receptor tyrosine kinase 3
ERCC1: ERCC excision repair 1,endonuclease non-catalytic subunit
ERCC6: ERCC excision repair 6,chromatin remodeling factor
ERGIC3: ERGIC and golgi 3
ESR1: Estrogen receptor 1
ESR2: Estrogen receptor 2
ESRRA: Estrogen related receptorα
ETS2: ETS proto-oncogene 2,transcription factor
ETV4: ETS variant transcription factor 4
EVL: Enah/Vasp-like
EU: European Union
EXO1: Exonuclease 1
EZH2: Enhancer of zeste 2 polycomb repressive complex 2 subunit
F3: Coagulation factor III, tissue factor
FABP7: Fatty acid binding protein 7
FAS: Fas cell surface death receptor
FASLG: Fas ligand
FASN: Fatty acid synthase
FBL: Fibrillarin
FBXW7: F-box and WD repeat domain containing 7
FEN1: Flap structure-specific endonuclease 1
FGD5: FYVE, RhoGEF and PH domain containing 5
FGF10: Fibroblast growth factor 10
FGF3: Fibroblast growth factor 3
FGF4: Fibroblast growth factor 4
FGF9: Fibroblast growth factor 9
FGFR1: Fibroblast growth factor receptor 1
FGFR2: Fibroblast growth factor receptor 2
FHIT: Fragile histidine triad diadenosine triphosphatase
FHL2: Four and a half LIM domains 2
FKBPL: FKBP prolyl isomerase like
FLACC1: Flagellum associated containing coiled-coil domains 1
FLNA: Folliculin
FLT1: Fms related receptor tyrosine kinase 1
FN1: Fibronectin 1
FOS: Fos proto-oncogene, AP-1 transcription factor subunit
FOSB: FosB proto-oncogene, AP-1 transcription factor subunit
FOSL2: FOS like 2,AP-1 transcription factor subunit
FOXA1x: Forkhead box A1
FOXM1: Forkhead box M1
FOXP3: Forkhead box P3
FOXQ1: Forkhead box Q1
FST: Follistatin
FTO: FTO alpha-ketoglutarate dependent dioxygenase
FUBP1: Far upstream element binding protein 1
GALNT16: Polypeptide N-acetylgalactosaminyltransferase 16
GAST: Gastrin
GATA6: GATA binding protein 6
GC: GC vitamin D binding protein
GCLC: Glutamate-cysteine ligase catalytic subunit
GDF10: Growth differentiation factor 10
GEO: Gene Expression Omnibus
GJA1: Gap junction protein α 1
GJB1: Gap junction protein beta 1
GNAI2: G protein subunit α i2
GNMT: Glycine N-methyltransferase
GPER1: G protein-coupled estrogen receptor 1
GPI: Glucose-6-phosphate isomerase
GPNMB: Glycoprotein nmb
GPX1: Glutathione peroxidase 1
GPX2: Glutathione peroxidase 2
GPX3: Glutathione peroxidase 3
GPX4: Glutathione peroxidase 4
GRB7: Growth factor receptor bound protein 7
GRIK2: Glutamate ionotropic receptor kainate type subunit 2
GSK3B: Glycogen synthase kinase 3β
GSTM1: Glutathione S-transferase mu 1
GSTP1: Glutathione S-transferase pi 1
GSTP2: Glutathione S-transferase, pi 2
GSTT1: Glutathione S-transferase theta 1
GUCY1A2: Guanylate cyclase 1 soluble subunit α 2
GZMB: Granzyme B
H1-2: H1.2 linker histone, cluster member
H19: H19 imprinted maternally expressed transcript
H2AX: H2A.X variant histone
H2BC12: H2B clustered histone 12
H2BC4: H2B clustered histone 4
H6PD: Hexose-6-phosphate dehydrogenase/glucose 1-dehydrogenase
HADHB: Hydroxyacyl-CoA dehydrogenase trifunctional multienzyme complex subunitβ
HAPLN4: Hyaluronan and proteoglycan link protein 4
HES1: Hes family bHLH transcription factor 1
HEY1: Hes related family bHLH transcription factor with YRPW motif 1
HEY2: Hes related family bHLH transcription factor with YRPW motif 2
HEYL: Hes related family bHLH transcription factor with YRPW motif like
HHEX: Hematopoietically expressed homeobox
HIC1: HIC ZBTB transcriptional repressor 1
HIF1A: Hypoxia inducible factor 1 subunit alpha
HILPDA: Hypoxia inducible lipid droplet associated
HMMR: Hyaluronan mediated motility receptor
HMOX1: Heme oxygenase 1
HNRNPK: Heterogeneous nuclear ribonucleoprotein K
HNRNPL: Heterogeneous nuclear ribonucleoprotein L
HNRNPR: Heterogeneous nuclear ribonucleoprotein R
HOXB13: Homeobox B13
HOXB9: Homeobox B9
HOXD11: Homeobox D11
HP: Haptoglobin
HPSE: Heparinase
HRAS: HRas proto-oncogene, GTPase
HRG: Histidine rich glycoprotein
HSP90AA1: Heat shock protein 90 alpha family class A member 1
HSPA1B: Heat shock protein family A (Hsp70) member 1B
HTRA1: HtrA serine peptidase 1
IARC: International Agency for Research in Cancer
lIBSP: Integrin binding sialoprotein
ICAM5: Intercellular adhesion molecule 5
ID3: Inhibitor of DNA binding 3
IDO1: Indoleamine 2,3-dioxygenase 1
IDS: Iduronate 2-sulfatase
IER2: Immediate early response 2
IFNB1: Interferon β 1
IFNG: Interferon gamma
IGBP1: Immunoglobulin binding protein 1
IGF1: Insulin like growth factor 1
IGF1R: Insulin like growth factor 1 receptor
IGFBP5: Insulin like growth factor binding protein 5
IGFBP7: Insulin like growth factor binding protein 7
IKBKG: Inhibitor of nuclear factor kappa B kinase regulatory subunit gamma
IL10: Interleukin 10
IL1B: Interleukin 1β
IL1R2: Interleukin 1 receptor type 2
IL2: Interleukin 2
IL24: Interleukin 24
IL6: Interleukin 6
IQSEC1: IQ motif and Sect. 7 domain ArfGEF 1
IRF1: Interferon regulatory factor 1
IRF4: Interferon regulatory factor 4
ITSN2: Intersectin 2
JAG1: Jagged canonical Notch ligand 1
JAG2: Jagged canonical Notch ligand 2
JMJD6: Jumonji domain containing 6
JUN: Jun proto-oncogene
JUNB: JunB proto-oncogene
JUND: JunD proto-oncogene
KCNH1: Potassium voltage-gated channel subfamily H member 1
KDR: Kinase insert domain receptor
KIT: KIT proto-oncogene
KLHDC10: Kelch domain containing 10
KLHDC7A: Kelch domain containing 7 A
KLK10: Kallikrein related peptidase 10
KMT2D: Lysine methyltransferase 2D
KRAS: KRAS proto-oncogene
KRT14: Keratin 14
KRT18: Keratin 18
KRT5: Keratin 5
KRT8: Keratin 8
L3MBTL3: L3MBTL histone methyl-lysine binding protein 3
LAMTOR5: Late endosomal/lysosomal adaptor, MAPK and MTOR activator 5
LBX1: Ladybird homeobox 1
LDHAL6B: Lactate dehydrogenase A like 6B
LDHB: Lactate dehydrogenase B
LECT2: Leukocyte cell derived chemotaxin 2
LEF1: Lymphoid enhancer binding factor 1
LEP: Leptin
LEPR: Leptin receptor
LGR6: Leucine rich repeat containing G protein-coupled receptor 6
LIMD2: LIM domain containing 2
LINC00115: Long intergenic non-protein coding RNA 115
LINC00671: Long intergenic non-protein coding RNA 671
LLGL1: LLGL scribble cell polarity complex component 1
LLGL2: LLGL scribble cell polarity complex component 2
LMNTD1: Lamin tail domain containing 1
LOXL2: Lysyl oxidase like 2
LPAR1: Lysophosphatidic acid receptor 1
LRRC37A: Leucine rich repeat containing 37 A
LRRC3B: Leucine rich repeat containing 3B
LSP1: Lymphocyte specific protein 1
MACIR: macrophage immunometabolism regulator
MAL: Mal
MALAT1: Metastasis associated lung adenocarcinoma transcript 1
MAN2C1: Mannosidase alpha class 2 C member 1
MAP2K7: Mitogen-activated protein kinase kinase 7
MAP3K1: Mitogen-activated protein kinase kinase kinase 1
MAP3K8: Mitogen-activated protein kinase kinase kinase 8
MAP4K4: Mitogen-activated protein kinase kinase kinase kinase 4
MAPK1: Mitogen-activated protein kinase 1
MAPK14: Mitogen-activated protein kinase 14
MAPK3: Mitogen-activated protein kinase 3
MARCKS: Myristoylated alanine rich protein kinase C substrate
MCL1: MCL1 apoptosis regulator
MDM2: MDM2 proto-oncogene
MDM4: MDM4 regulator of p53
MECOM: MDS1 and EVI1 complex locus
MED12: Mediator complex subunit 12
MED28: Mediator complex subunit 28
MEIS1: Meis homeobox 1
MET: MET proto-oncogen
METTL6: Methyltransferase 6
MFGE8: Milk fat globule EGF and factor V/VIII domain containing
MIF: Macrophage migration inhibitory factor
MIR10A: MicroRNA 10a
MIR1246: MicroRNA 1246
MIR126: MicroRNA 126
MIR127: MicroRNA 127
MIR136: MicroRNA 136
MIR141: MicroRNA 141
MIR145: MicroRNA 145
MIR146A: MicroRNA 146a
MIR152: MicroRNA 152
MIR154: MicroRNA 154
MIR155: MicroRNA 155
MIR193A: MicroRNA 193a
MIR200B: MicroRNA 200b
MIR200C: MicroRNA 200c
MIR205: MicroRNA 205
MIR206: MicroRNA 206
MIR21: MicroRNA 21
MIR22: MicroRNA 22
MIR221: MicroRNA 221
MIR222: MicroRNA 222
MIR224: MicroRNA 224
MIR242: MicroRNA 242
MIR24-2: MicroRNA 24 − 2
MIR29A: MicroRNA 29a
MIR30: MicroRNA 30
MIR301A: MicroRNA 301a
MIR302D: MicroRNA 302d
MIR30A: MicroRNA 30a
MIR31: MicroRNA 31
MIR31HG: MIR31 host gene
MIR34B: MicroRNA 34b
MIR34C: MicroRNA 34c
MIR369: MicroRNA 369
MIR370: MicroRNA 370
MIR410: MicroRNA 410
MIR429: MicroRNA 429
MIR4435-2HG: MIR4435-2 host gene
MIR487B: MicroRNA 487b
MIR494: MicroRNA 494
MIR506: MicroRNA 506
MIR98: MicroRNA 98
MIRLET7BHG: MIRLET7B host gene
MKI67: Marker of proliferation Ki-67
MME: Membrane metalloendopeptidase
MMP1: Matrix metallopeptidase 1
MMP1: Matrix metallopeptidase 1
MMP10: Matrix metallopeptidase 10
MMP14: Matrix metallopeptidase 14
MMP1A: Matrix metallopeptidase 1a
MMP2: Matrix metallopeptidase 2
MMP3: Matrix metallopeptidase 3
MMP9: Matrix metallopeptidase 9
MOE: South Korea Ministry of Environment
MOL: South Korea Ministry of Labour
MPO: Myeloperoxidase
MPP1: MAGUK p55 scaffold protein 1
MRPL13: Mitochondrial ribosomal protein L13
MRPL19: Mitochondrial ribosomal protein L19
MRPL9: Mitochondrial ribosomal protein L9
MRPS22: Mitochondrial ribosomal protein S22
MRPS23: Mitochondrial ribosomal protein S23
MRPS28: Mitochondrial ribosomal protein S28
MRPS7: Mitochondrial ribosomal protein S7
MST1: Macrophage stimulating 1
MT3: Metallothionein 3
MTDH: Metadherin
MTHFR: Methylenetetrahydrofolate reductase
MTOR: Mechanistic target of rapamycin kinase
MTR: 5-methyltetrahydrofolate-homocysteine methyltransferase
MUC12: Mucin 12
MUC16: Mucin 16
MYC: MYC proto-oncogene
MYH9: Myosin heavy chain 9
MYO18B: Myosin XVIIIB
NAT2: N-acetyltransferase 2
NCOA1: Nuclear receptor coactivator 1
NCOA2: Nuclear receptor coactivator 2
NCOA3: Nuclear receptor coactivator 3
NCOR1: Nuclear receptor corepressor 1
NDRG1: N-myc downstream regulated 1
NDUFS3: NADH: ubiquinone oxidoreductase core subunit S3
NECTIN2: Nectin cell adhesion molecule 2
NFE2L2: NFE2 like bZIP transcription factor 2
NFKBIA: NFKB inhibitorα
NFYA: Nuclear transcription factor Y subunitα
NISCH: Nischarin
NMBR: Neuromedin B receptor
NOP9: NOP9 nucleolar protein
NOS2: Nitric oxide synthase 2
NOS3: Nitric oxide synthase 3
NOTCH1: Notch receptor 1
NOTCH2: Notch receptor 2
NOTCH3: Notch receptor 3
NOTCH4: Notch receptor 4
NPPA: Natriuretic peptide A
NQO1: NAD(P)H quinone dehydrogenase 1
NQO1: NAD(P)H quinone dehydrogenase 1
NQO2: NAD(P)H quinone dehydrogenase 2
NR2F1: Nuclear receptor subfamily 2 group F member 1
NR2F6: Nuclear receptor subfamily 2 group F member 6
NR2F6: Nuclear receptor subfamily 2 group F member 6
NRCAM: Neuronal cell adhesion molecule
NRG1: Neuregulin 1
NRIP1: Nuclear receptor interacting protein 1
NSD2: Nuclear receptor binding SET domain protein 2
NSUN6: NOP2/Sun RNA methyltransferase 6
NUDT17: Nudix hydrolase 17
NUDT2: Nudix hydrolase 2
O&G: Oil and gas
OCLN: Occluding
OEHHA: Office of Environment Health Hazard Assessment
OGG1: 8-oxoguanine DNA glycosylase
PABPC1: Poly(A) binding protein cytoplasmic 1
PAEP: Progestagen associated endometrial protein
PAK1: P21 (RAC1) activated kinase 1
PALB2: Partner and localizer of BRCA2
PARP1: Poly(ADP-ribose) polymerase 1
PCBP1: Poly(rC) binding protein 1
PCDHGB6: Protocadherin gamma subfamily B,6
PCNA: Proliferating cell nuclear antigen
PDCD1: Programmed cell death 1
PDCD4: Programmed cell death 4
PDE2A: Phosphodiesterase 2 A
PDGFA: Platelet derived growth factor subunit A
PDLIM4: PDZ and LIM domain 4
PDPK1: 3-phosphoinositide dependent protein kinase 1
PDZK1: PDZ domain containing 1
PER3: Period circadian regulator 3
PGGT1B: Protein geranylgeranyltransferase type I subunit beta
PGR: Progesterone receptor
PHB1: Prohibitin 1
PHGDH: Phosphoglycerate dehydrogenase
PIK3CA: Phosphatidylinositol-4,5-bisphosphate 3-kinase catalytic subunitα
PIM1: Pim-1 proto-oncogene, serine/threonine kinase
PIN1: Peptidylprolyl cis/trans isomerase, NIMA-interacting 1
PLA2G4A: Phospholipase A2 group IVA
PLEKHD1: Pleckstrin homology and coiled-coil domain containing D1
PON1: Paraoxonase 1
PPARGC1B: PPARG coactivator 1β
PPBP: Pro-platelet basic protein
PPM1D: Protein phosphatase, Mg2+/Mn2 + dependent 1D
PPP1R12B: Protein phosphatase 1 regulatory subunit 12B
PPP2R1B: Protein phosphatase 2 scaffold subunit 1β
PPRTV: USEPA Provisional Peer-Reviewed Toxicity Values
PRC1: Protein regulator of cytokinesis 1
PRDX1: Peroxiredoxin 1
PRDX6: Peroxiredoxin 6
PRKN: Parkin RBR E3 ubiquitin protein ligase
PRSS46: Protease, serine 46
PSMA4: Proteasome 20 S subunit α 4
PTEN: Phosphatase and tensin homolog
PTGIS: Prostaglandin I2 synthase
PTGS1: Prostaglandin-endoperoxide synthase 1
PTGS2: Prostaglandin-endoperoxide synthase 2
PTHLH: Parathyroid hormone like hormone
PTMA: Prothymosinα
PTPRD: Protein tyrosine phosphatase receptor type D
PYCARD: PYD and CARD domain containing
RAD51: RAD51 recombinase
RAD51B: RAD51 paralog B
RAD51C: RAD51 paralog C
RAD52: RAD52 homolog, DNA repair protein
RAD54L: RAD54 like
RAF1: Raf-1 proto-oncogene, serine/threonine kinase
RALYL: RALY RNA binding protein like
RAMP2: Receptor activity modifying protein 2
RARA: Retinoic acid receptorα
RARB: Retinoic acid receptorβ
RASSF1: Ras association domain family member 1
RB1: RB transcriptional corepressor 1
RB1CC1: RB1 inducible coiled-coil 1
RBM3: RNA binding motif protein 3
RBP4: Retinol binding protein 4
RCCD1: RCC1 domain containing 1
RCHY1: Ring finger and CHY zinc finger domain containing 1
RECQL: RecQ like helicase
RELA: RELA proto-oncogene, NF-kB subunit
REPS2: RALBP1 associated Eps domain containing 2
RGS2: Regulator of G protein signaling 2
RIBC2: RIB43A domain with coiled-coils 2
RIC8A: RIC8 guanine nucleotide exchange factor A
RIOX2: Ribosomal oxygenase 2
RMND1: Required for meiotic nuclear division 1 homolog
RNASET2: Ribonuclease T2
RNF115: Ring finger protein 115
RNF182: Ring finger protein 182
ROBO1: Roundabout guidance receptor 1
ROR1: Receptor tyrosine kinase like orphan receptor 1
RPL23A: Ribosomal protein L23a
RPL31: Ribosomal protein L31
RPLP2: Ribosomal protein lateral stalk subunit P2
RPS4X: Ribosomal protein S4 X-linked
RPS6: Ribosomal protein S6
RPS6KB2: Ribosomal protein S6 kinase B2
RPS7: Ribosomal protein S7
RPS8: Ribosomal protein S8
RRAD: Ras related glycolysis inhibitor and calcium channel regulator
RSPO3: R-spondin 3
RTEL1: Regulator of telomere elongation helicase 1
RUNX2: RUNX family transcription factor 2
RUNX3: RUNX family transcription factor 3
RXRB: Retinoid X receptorβ
SECISBP2L: SECIS binding protein 2 like
SELENBP1: Selenium binding protein 1
SELENOP: Selenoprotein P
SERPINA1: Serpin family A member 1
SERPINB2: Serpin family B member 2
SERPINB5: Serpin family B member 5
SERPING1: Serpin family G member 1
SETBP1: SET binding protein 1
SETD2: SET domain containing 2,histone lysine methyltransferase
SFRP1: Secreted frizzled related protein 1
SFRP2: Secreted frizzled related protein 2
SFRP5: Secreted frizzled related protein 5
SFTPB: Surfactant protein B
SFTPD: Surfactant protein D
SHMT1: Serine hydroxymethyltransferase 1
SIDT2: SID1 transmembrane family member 2
SIM1: SIM bHLH transcription factor 1
SIRT1: Sirtuin 1
SLC10A6: Solute carrier family 10 member 6
SLC16A3: Solute carrier family 16 member 3
SLC22A18: Solute carrier family 22 member 18
SLC28A1: Solute carrier family 28 member 1
SLC2A1: Solute carrier family 2 member 1
SLC2A10: Solute carrier family 2 member 10
SLC2A2: Solute carrier family 2 member 2
SLC2A5: Solute carrier family 2 member 5
SLC39A6: Solute carrier family 39 member 6
SLC3A2: Solute carrier family 3 member 2
SLC5A5: Solute carrier family 5 member 5
SLC7A5: Solute carrier family 7 member 5
SLCO1B1: Solute carrier organic anion transporter family member 1B1
SLCO1B3: Solute carrier organic anion transporter family member 1B3
SMARCC1: SWI/SNF related, matrix associated, actin dependent regulator of chromatin subfamily c member 1
SMC2: Structural maintenance of chromosomes 2
SNAI1: Snail family transcriptional repressor 1
SNAI2: Snail family transcriptional repressor 2
SNCG: Synuclein gamma
SND1: Staphylococcal nuclease and tudor domain containing 1
SNX32: Sorting nexin 32
SOD2: Superoxide dismutase 2
SOX2: SRY-box transcription factor 2
SOX30: SRY-box transcription factor 30
SOX9: SRY-box transcription factor 9
SPATA18: Spermatogenesis associated 18
SPP1: Secreted phosphoprotein 1
SPRY2: Sprouty RTK signaling antagonist 2
SRC: SRC proto-oncogene, non-receptor tyrosine kinase
SREBF2: Sterol regulatory element binding transcription factor 2
STARD8: StAR related lipid transfer domain containing 8
STAT3: Signal transducer and activator of transcription 3
STAT5A: Signal transducer and activator of transcription 5 A
STC2: Stanniocalcin 2
STIM1: Stromal interaction molecule 1
STK11: Serine/threonine kinase 11
STMN1: Stathmin 1
STN1: STN1 subunit of CST complex
STXBP4: Syntaxin binding protein 4
SULT1A1: Sulfotransferase family 1 A member 1
SYNE1: Spectrin repeat containing nuclear envelope protein 1
SYNJ2: Synaptojanin 2
TAFA4: TAFA chemokine like family member 4
TANK: TRAF family member associated NFKB activator
TBX3: T-box transcription factor
TCL1B: TCL1 family AKT coactivator B
TEP1: Telomerase associated protein 1
TERT: Telomerase reverse transcriptase
TFAP2A: Transcription factor AP-2α
TFPI2: Tissue factor pathway inhibitor 2
TFRC: Transferrin receptor
TGFB1: Transforming growth factor β 1
TGFBR2: Transforming growth factor beta receptor 2
TGM2: Transglutaminase 2
THBS1: Thrombospondin 1
THEMIS2: Thymocyte selection associated family member 2
TLE3: TLE family member 3
TLR4: Toll like receptor 4
TMEM25: Transmembrane protein 25
TMEM45A: Transmembrane protein 45 A
TNF: Tumor necrosis factor
TNFSF10: TNF superfamily member 10
TNIP1: TNFAIP3 interacting protein 1
TOP2A: DNA topoisomerase IIα
TOX3: TOX high mobility group box family member 3
TP53: Tumor protein p53
TP53BP1: Tumor protein p53 binding protein 1
TP53BP2: Tumor protein p53 binding protein 2
TP63: Tumor protein p63
TP73: Tumor protein p73
TRERF1: Transcriptional regulating factor 1
TRIM12A: Tripartite motif-containing 12 A
TRIM47: Tripartite motif containing 47
TRIO: Trio Rho guanine nucleotide exchange factor
TRMT11: TRNA methyltransferase 11 homolog
TRP53: Transformation related protein 53
TRP63: Transformation related protein 63
TSC2: TSC complex subunit 2
TSHR: Thyroid stimulating hormone receptor
TTR: Transthyretin
TUBB3: Tubulin beta 3 class III
TXN: Thioredoxin
TYMS: Thymidylate synthetase
TYRP1: Tyrosinase related protein 1
UBD: Ubiquitin D
UBE2C: Ubiquitin conjugating enzyme E2 C
UBLCP1: Ubiquitin like domain containing CTD phosphatase 1
UGT2B17: UDP glucuronosyltransferase family 2 member B17
UMPS: Uridine monophosphate synthetase
UN GHS: United Nations Globally Harmonized System of Classification and Labelling of Chemicals
UPK1B: Uroplakin 1B
USP18: Ubiquitin specific peptidase 18
VDR: Vitamin D receptor
VEGFB: Vascular endothelial growth factor B
VEGFC: Vascular endothelial growth factor C
VHL: Von Hippel-Lindau tumor suppressor
VIM: Vimentin
VPS39: VPS39 subunit of HOPS complex
WNT10B: Wnt family member 10B
WNT5A: Wnt family member 5 A
WT1: WT1 transcription factor
WWOX: WW domain containing oxidoreductase
XPC: XPC complex subunit
XRCC2: X-ray repair cross complementing 2
XRCC3: X-ray repair cross complementing 3
YAP1: Yes1 associated transcriptional regulator
YBX1: Y-box binding protein 1
ZC3H11A: Zinc finger CCCH-type containing 11 A
ZEB1: Zinc finger E-box binding homeobox 1
ZEB2: Zinc finger E-box binding homeobox 2
ZFP366: Zinc finger protein 366
ZNF365: Zinc finger protein 365
ZNF366: Zinc finger protein 366
ZNF404: Zinc finger protein 404
ZNF432: Zinc finger protein 432
ZNF595: Zinc finger protein 595
ZSCAN22: Zinc finger and SCAN domain containing 22
ZSWIM5: Zinc finger SWIM-type containing 5
