# Supplementary material for: Mapping the influence of hydrocarbons mixture on molecular mechanisms, involved in breast and lung neoplasms: in silico toxicogenomic data-mining
Source: Genes Environ. 2024 Jul 9;46:15. doi: 10.1186/s41021-024-00310-y (PMC11232146; doi:10.1186/s41021-024-00310-y)
Supplement: Supplementary file 1 — Supplementary Material 1 [file 41021_2024_310_MOESM1_ESM.docx]

**Supplementary Table 1:** Carcinogens and their GHS classifications

| **No** | **CAS number** | **Chemical name** | **Most stringent carcinogenicity classification among 9 databases (Ismail, 2019)^1^** | **Most stringent carcinogenicity classification among 6 database (This study, 2023)^2^** | **Hydrocarbon^3^** |
| --- | --- | --- | --- | --- | --- |
| 1 | 79-34-5 | 1,1,2,2-Tetrachloroethane^*^ | 2 | 1B | Yes |
| 2 | 79-00-5 | 1,1,2-Trichloroethane | 2 | 2 | Yes |
| 3 | 75-34-3 | 1,1-Dichloroethane | 2 | Not classified | Yes |
| 4 | 75-35-4 | 1,1-Dichloroethylene | 2 | 2 | Yes |
| 5 | 106-93-4 | 1,2-Dibromoethane | 1A | 1B | Yes |
| 6 | 107-06-2 | 1,2-Dichloroethane | 1A | 1B | Yes |
| 7 | 78-87-5 | 1,2-Dichloropropane | 1B | 1A | Yes |
| 8 | 106-99-0 | 1,3-Butadiene | 1A | 1A | Yes |
| 9 | 542-75-6 | 1,3-Dichloropropene | 2 | 2 | Yes |
| 10 | 106-46-7 | 1,4-Dichlorobenzene | 2 | 2 | Yes |
| 11 | 123-91-1 | 1,4-Dioxane | 1A | 1B | No |
| 12 | 78-79-5 | 2-Methyl-1,3-butadiene | 1A | 1A | Yes |
| 13 | 75-07-0 | Acetaldehyde | 1B | 1B | No |
| 14 | 62-53-3 | Aniline^*^ | 2 | 1B | No |
| 15 | 120-12-7 | Anthracene^*^ | 2 | 1B | Yes |
| 16 | 7440-36-0 | Antimony | 2 | 2 | No |
| 17 | 7440-38-2 | Arsenic | 1A | 1 | No |
| 18 | 1332-21-4 | Asbestos | 1A | 1 | No |
| 19 | 71-43-2 | Benzene | 1A | 1 | Yes |
| 20 | 56-55-3 | Benzo(a)anthracene | 1B | 1B | Yes |
| 21 | 50-32-8 | Benzo(a)pyrene | 1A | 1A | Yes |
| 22 | 205-99-2 | Benzo(b)fluoranthene | 1B | 1B | Yes |
| 23 | 192-97-2 | Benzo(e)pyrene | 1B | 1B | Yes |
| 24 | 207-08-9 | Benzo(k)fluoranthene | 1B | 1B | Yes |

| **No** | **CAS number** | **Chemical name** | **Most stringent carcinogenicity classification among 9 databases (Ismail, 2019)^1^** | **Most stringent carcinogenicity classification among 6 database (This study, 2023)^2^** | **Hydrocarbon^3^** |
| --- | --- | --- | --- | --- | --- |
| 25 | 7440-41-7 | Beryllium | 1A | 1 | No |
| 26 | 92-52-4 | Biphenyl^*^ | 2 | 1B | Yes |
| 27 | 117-81-7 | Bis(2-ethyl hexyl) phthalate | 1B | 1B | No |
| 28 | 7440-43-9 | Cadmium | 1A | 1A | No |
| 29 | 56-23-5 | Carbon tetrachloride | 1B | 1B | Yes |
| 30 | 108-90-7 | Chlorobenzene | 2 | 2 | Yes |
| 31 | 75-00-3 | Chloroethane | 2 | 2 | Yes |
| 32 | 67-66-3 | Chloroform | 1B | 2 | Yes |
| 33 | 74-87-3 | Chloromethane | 2 | 2 | Yes |
| 34 | 18540-29-9 | Chromium (hexavalent) | 1A | 1 | No |
| 35 | 218-01-9 | Chrysene | 1A | 1B | Yes |
| 36 | 7440-48-4 | Cobalt | 1B | 1B | No |
| 37 | 1319-77-3 | Cresols (total) | 2 | 2 | No |
| 38 | 98-82-8 | Cumene | 1B | 1B | Yes |
| 39 | 53-70-3 | Dibenz(a,h)anthracene | 1B | 1B | Yes |
| 40 | 75-09-2 | Dichloromethane^*^ | 2 | 1A | Yes |
| 41 | 111-42-2 | Diethanolamine | 2 | 2 | No |
| 42 | 100-41-4 | Ethylbenzene | 2 | 2 | Yes |
| 43 | 50-00-0 | Formaldehyde | 1A | 1A | No |
| 44 | 57117-31-4 | Furan:5F 23478k | 1A | Not classified | No |
| 45 | 67-72-1 | Hexachloroethane | 2 | 2 | Yes |
| 46 | 1333-74-0 | Hydrogen | 1A | Not classified | No |
| 47 | 193-39-5 | Indeno(1,2,3-cd)pyrene | 2 | 2 | Yes |
| 48 | 75-28-5 | Isobutane | 1A | 1A | Yes |

| **No** | **CAS number** | **Chemical name** | **Most stringent carcinogenicity classification among 9 databases (Ismail, 2019)^1^** | **Most stringent carcinogenicity classification among 6 databases (This study, 2023)^2^** | **Hydrocarbon^3^** |
| --- | --- | --- | --- | --- | --- |
| 49 | 7439-92-1 | Lead | 2 | 2 | No |
| 50 | 108-39-4 | m-Cresol | 2 | 2 | No |
| 51 | 108-10-1 | Methyl isobutyl ketone^*^ | 2 | 1B | No |
| 52 | 1634-04-4 | Methyl tert-butyl ether | 2 | Not classified | No |
| 53 | 91-20-3 | Naphthalene | 2 | 2 | Yes |
| 54 | 106-97-8 | n-Butane (containing ≥ 0.1% butadiene) | 1A | 1A | Yes |
| 55 | 7440-02-0 | Nickel | 2 | 2 | No |
| 56 | 95-48-7 | o-Cresol | 2 | 2 | No |
| 57 | 106-44-5 | p-Cresol | 2 | 2 | No |
| 58 | 198-55-0 | Perylene | 2 | Not classified | Yes |
| 59 | 1336-36-3 | Polychlorinated biphenyls (total) | 1B | 1 | Yes |
| 60 | 74-98-6 | Propane | 1A | Not classified | Yes |
| 61 | 75-56-9 | Propylene oxide | 1A | 1B | No |
| 62 | 100-42-5 | Styrene^*^ | 2 | 1B | Yes |
| 63 | 1746-01-6 | Tetrachlorodibenzodioxin | 1A | 1A | No |
| 64 | 127-18-4 | Tetrachloroethylene | 1A | 1B | Yes |
| 65 | 79-01-6 | Trichloroethylene | 1A | 1A | Yes |
| 66 | 593-60-2 | Vinyl bromide | 1A | 1B | Yes |
| 67 | 75-01-4 | Vinyl chloride | 1A | 1A | Yes |

^1^The 9 reference databases are Australian Hazardous Chemical Information System ([Safe Work Australia - Search Hazardous Chemicals](https://hcis.safeworkaustralia.gov.au/HazardousChemical)), China ([ChemRadar - Stay Ahead of Chemical Compliance Risks in China and APAC | By CIRS Group](https://www.chemradar.com/)), European Chemicals Agency ([Homepage - ECHA (europa.eu)](https://echa.europa.eu/)), Japan National Institute of Technology and Evaluation (NITE) ([National Institute of Technology and Evaluation (NITE)](https://www.nite.go.jp/en/)), Malaysia Department of Occupational Safety and Health ([Official Website Department of Occupational Safety and Health - Chemical Management (dosh.gov.my)](https://www.dosh.gov.my/index.php/legislation/codes-of-practice/chemical-management)), Myanmar ([Search - AJCSD (ASEAN-Japan Chemical Safety Database)](https://www.ajcsd.org/chrip_search/srhInput)), New Zealand ([Chemical Classification and Information Database (CCID) | EPA](https://www.epa.govt.nz/database-search/chemical-classification-and-information-database-ccid/)), South Korea Ministry of Environment([Chemical Information Processing System (me.go.kr)](https://kreach.me.go.kr/repwrt/index.do)), South Korea Ministry of Labour (https://msds.kosha.or.kr/msds/), and Turkey.

^2^The 6 databases are those tabulated in Table 1.

^3^Based on [The Comparative Toxicogenomics Database | CTD (ctdbase.org)](https://ctdbase.org/)

^*^Chemicals with the most stringent carcinogenic classification increased from Category 2 to Category 1/1B/1A
