# Supplementary material for: Mapping the influence of hydrocarbons mixture on molecular mechanisms, involved in breast and lung neoplasms: in silico toxicogenomic data-mining
Source: Genes Environ. 2024 Jul 9;46:15. doi: 10.1186/s41021-024-00310-y (PMC11232146; doi:10.1186/s41021-024-00310-y)
Supplement: Supplementary file 3 — Supplementary Material 3 [file 41021_2024_310_MOESM3_ESM.docx]

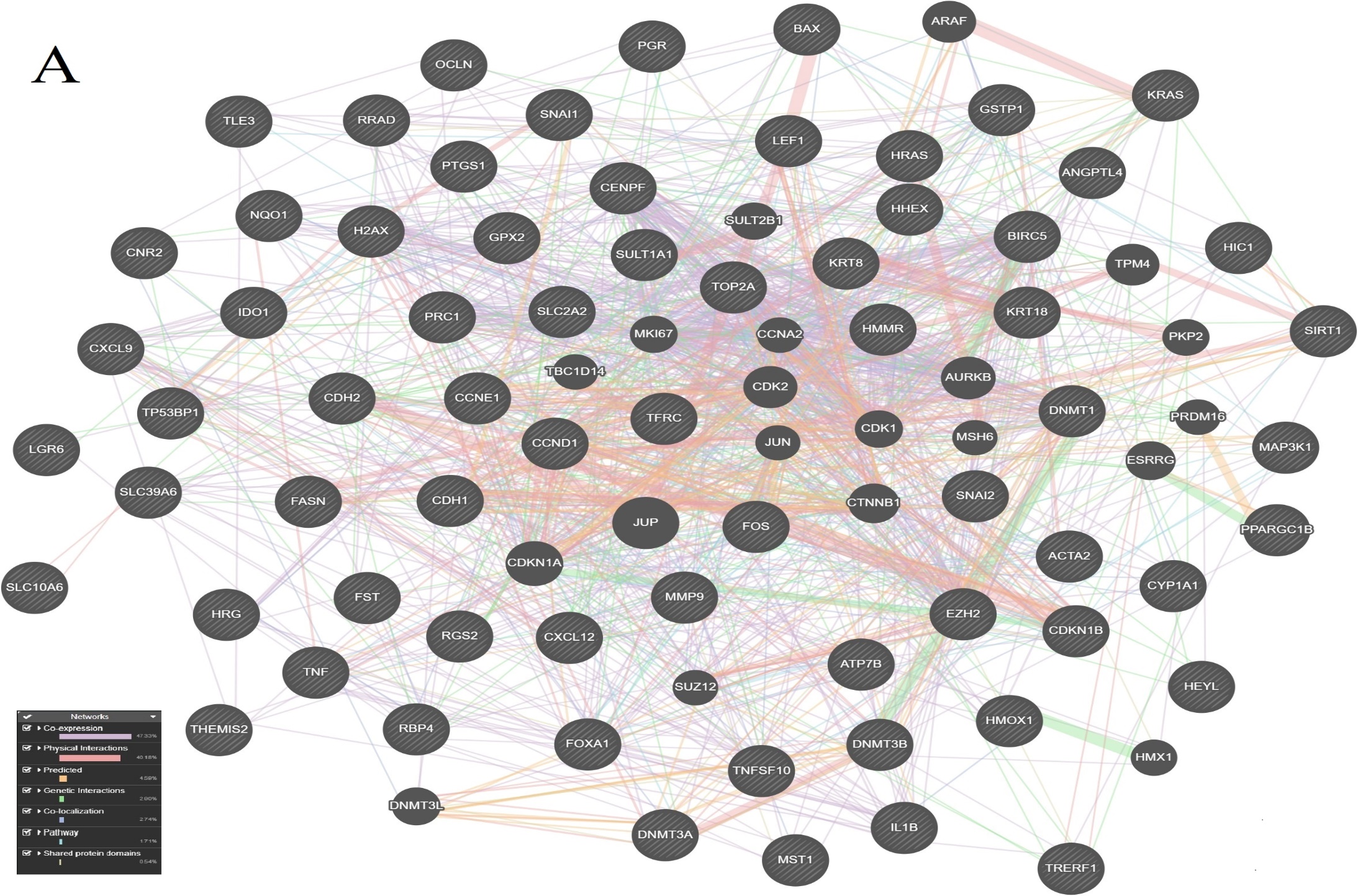


**
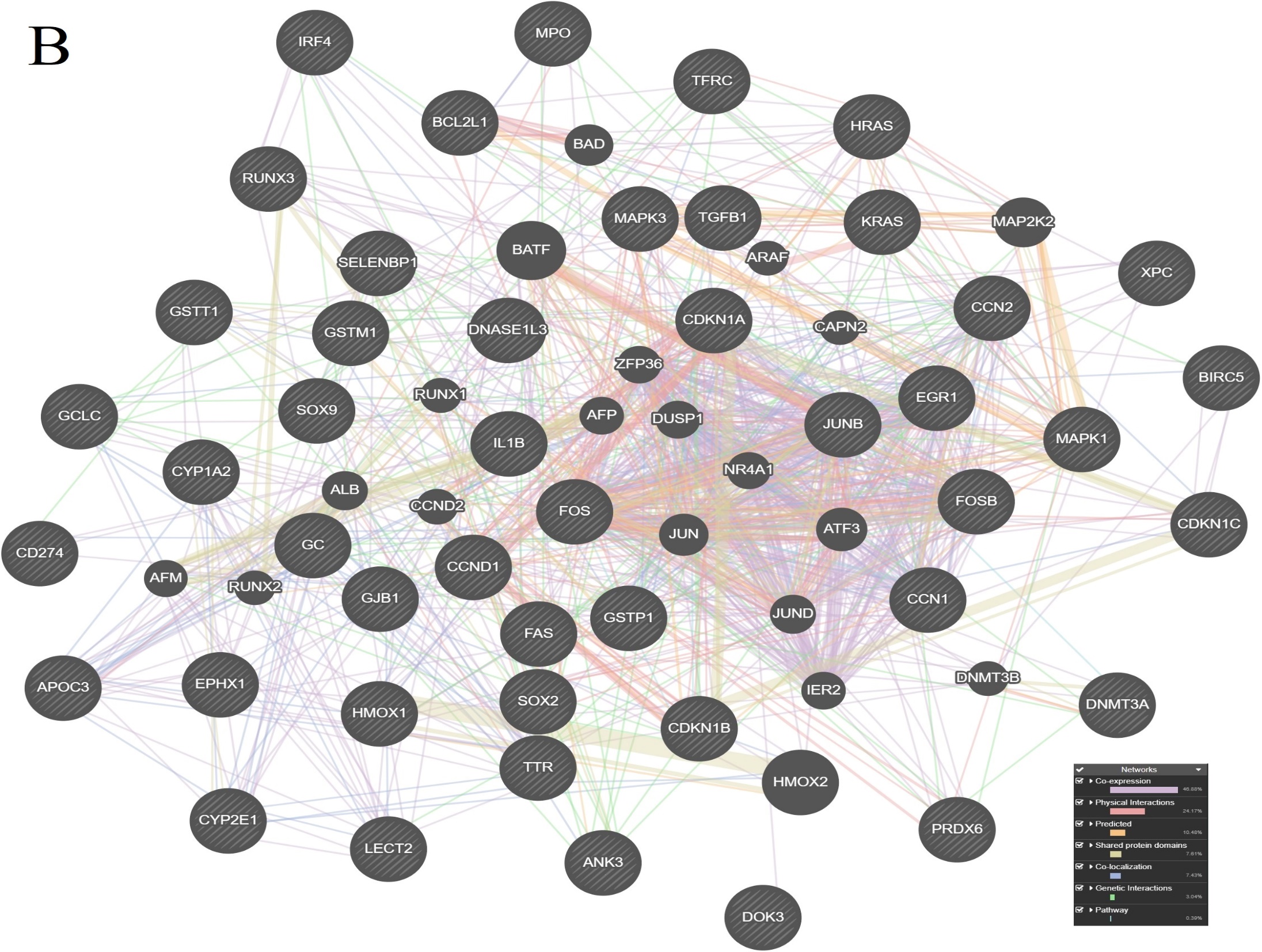
**

**Supplementary Figure 3:** Constructed network of overlapping genes affected by the investigated hydrocarbons along with genes related to breast and lung neoplasms. (A) Interaction network of 87 genes related to breast neoplasms affected by the investigated hydrocarbons. (B) Interaction network of 44 genes related to lung neoplasms affected by the investigated hydrocarbons (GeneMANIA, <https://genemania.org/>).
