# Supplementary material for: Mapping the influence of hydrocarbons mixture on molecular mechanisms, involved in breast and lung neoplasms: in silico toxicogenomic data-mining
Source: Genes Environ. 2024 Jul 9;46:15. doi: 10.1186/s41021-024-00310-y (PMC11232146; doi:10.1186/s41021-024-00310-y)
Supplement: Supplementary file 4 — Supplementary Material 4 [file 41021_2024_310_MOESM4_ESM.docx]

**Supplementary Table 2**: Genes connected to the selected hydrocarbons and linked to breast and lung neoplasms

| **Hydrocarbon Group** | | **Chemical Name** | | **CAS Number** | | **Breast Neoplasms Inference Network** | | **Inference Score & reference link** | **Lung Neoplasms Inference Network** | **Inference Score & reference link** |
| --- | --- | --- | --- | --- | --- | --- | --- | --- | --- | --- |
| Alkanes & alkenes | | Isobutane | | 75-28-5 | | No direct evidence on marker/mechanism of gene interaction | | - | No direct evidence on marker/mechanism of gene interaction | - |
|  |  | 2-Methyl-1,3-butadiene | | 78-79-5 | | **12 genes:** *CCND1 CCNE1 CDH1 CDH2 CDKN1B HRAS KRAS MIR141 MIR221 MIR29A OCLN SNAI2* | | 27  [isoprene ↔ Breast Neoplasms \| CTD (ctdbase.org)](https://ctdbase.org/detail.go?type=relationship&chemAcc=C005059&diseaseAcc=MESH%3AD001943&view=reference) | **8 genes:** *CCND1 CDKN1A CDKN1B HRAS KRAS MAPK1 MAPK3 MIR30A* | 19  [isoprene ↔ Lung Neoplasms \| CTD (ctdbase.org)](https://ctdbase.org/detail.go?type=relationship&chemAcc=C005059&diseaseAcc=MESH%3AD008175&view=reference) |
|  |  | n-Butane | | 106-97-8 | | No direct evidence on marker/mechanism of gene interaction | | - | No direct evidence on marker/mechanism of gene interaction | - |
|  |  | 1,3-Butadiene | | 106-99-0 | | **63 genes:** *ACTA2 ANGPTL4 ARID1A ATP7B BAX BIRC5 CENPF CNR2 CXCL12 CXCL9 CYP1A1 DNMT1 DNMT3A DNMT3B EZH2 FASN FOS FOXA1 FST GPX2 GSTP1 HEYL HHEX HIC1 HMMR HMOX1 HRAS HRG IDO1 IL1B KMT2D KRAS KRT18 LEF1 LGR6 MAP3K1 MIR145 MIR222 MIR242 MMP9 MST1 NQO1 PGR PPARGC1B PRC1 PTGS1 RBP4 RGS2 RRAD SIRT1 SLC10A6 SLC2A2 SLC39A6 SNAI1 SNAI2 SULT1A1 TFRC THEMIS2 TLE3 TNFSF10 TOP2A TRERF1 TRP53* | | 49  [1,3-butadiene ↔ Breast Neoplasms \| CTD (ctdbase.org)](https://ctdbase.org/detail.go?type=relationship&chemAcc=C031763&diseaseAcc=MESH%3AD001943&view=reference) | **48 genes:** *ANK3 APOC3 BCL2L1 BIRC5 CCN1 CCN2 CD274 CDKN1A CDKN1C CES1F CYP1A2 CYP2E1 DNASE1L3 DNMT3A DOK3 EGR1 EPHX1 FAS FOS FOSB GC GCLC GJB1 GSTM1 GSTP1 GSTP2 GSTT1 HMOX1 HRAS IL1B IRF4 JUNB KRAS LECT2 MIR136 MIR155 MIR222 MPO PRDX6 RUNX3 SELENBP1 SOX2 SOX9 TFRC TGFB1 TRP53 TTR XPC* | 46  [1,3-butadiene ↔ Lung Neoplasms \| CTD (ctdbase.org)](https://ctdbase.org/detail.go?type=relationship&chemAcc=C031763&diseaseAcc=MESH%3AD008175&view=reference) |
| Halogenated Hydrocarbon | | Carbon tetrachloride | | 56-23-5 | | No direct evidence on marker/mechanism of gene interaction | | - | **184 genes:** *A2M ACE ACSM1 ACTB ADA ADAM28 AKT1 ANK3 ANXA2 APOA1 APOC3 APOE ARHGEF5 AS3MT ATG101 ATOX1 AVPI1 AZGP1 BCL2L1 BECN1 BHLHE41 BIRC5 CASP8 CAV1 CCN1 CCN2 CCND1 CCNG1 CD274 CDH13 CDKN1A CDKN1B CDKN1C CDKN2A CEACAM1 CES1F CHEK2 CLPTM1L CLTB COX17 CPE CRP CTNNB1 CXCL14 CYP1B1 CYP2E1 DAB2IP DAPK1 DDR1 DNASE1L3 DNMT3A DOK1 DPYD EFEMP1 EFNB2 EGFR EGR1 EPHX1 ERBB2 ERBB3 ERCC1 ERGIC3 ESR1 FAS FASLG FEN1 FGF9 FGFR1 FGFR2 FHIT FOS FOSL2FOX M1 FUBP1 GAST GATA6 GC GCLC GJA1 GJB1 GPX1 GPX3 GRB7 GSTM1 GSTP1 GSTT1 HES1 HILPDA HMOX1 HRAS ID3 IER2 IFNG IKBKG IL10 IL1B IL1R2 IL2 IL6 IRF1 JAG1 JUN JUNB JUND KDR KRAS LECT2 MAP4K4 MAPK1 MAPK14 MAPK3 MARCKS MCL1 MET MIR10A MIR127 MIR136 MIR155 MIR21 MIR30 MIR31 MIR34C MIR370 MIR410 MMP1 MMP10 MPO MPP1 MTHFR MYC NOS2 NOTCH2 NOTCH3 NPPA NRG1 OGG1 PCNA PDCD4 PGGT1B PON1 PRDX1 PRDX6 PRKN PTEN PTGIS PTMA PYCARD RAD52 RAMP2 RARB RASSF1 RCHY1 ROBO1 SELENBP1 SELENOP SERPINA1 SERPING1 SIDT2 SLC22A18 SLC3A2 SLC7A5 SMC2 SND1 SOX9 SPP1 STIM1 STN1 TERT TFRC TGFB1 TGFBR2 TLR4 TMEM45A TNF TP53 TRP53 TSHR TTR TYMS UGT2B17 VHL WNT5A WT1 XPC* | 100  [Carbon Tetrachloride ↔ Lung Neoplasms \| CTD (ctdbase.org)](https://ctdbase.org/detail.go?type=relationship&chemAcc=D002251&diseaseAcc=MESH%3AD008175&view=reference)  Has associated exposure references |
|  | | Vinyl chloride | | 75-01-4 | | No direct evidence on marker/mechanism of gene interaction | | - | **16 genes:** *AKT1 CDKN2A CYP2E1 GSTM1 GSTP1 GSTT1 HMOX1 IFNG IL10 IL1B IL6 KRAS NOS2 TNF TP53 XPC* | 25  [Vinyl Chloride ↔ Lung Neoplasms \| CTD (ctdbase.org)](https://ctdbase.org/detail.go?type=relationship&chemAcc=D014752&diseaseAcc=MESH%3AD008175&view=reference)  Has associated exposure references |
|  | | Dichloromethane | | 75-09-2 | | **35 genes:** *AKT1 AR BCL2 BCL2A1 CAT CDH1 CTNNB1 CXCL8 CXCR4 CYP1A1 CYP1B1 ESR1 FOS FOXA1 GPX1 HMOX1 IFNG IL10 IL6 JUNMMP1 MMP9 NCOR1 NDUFS3 NFE2L2 NFKBIA NOS2NQO1NR2F6 PER3 PTGS2 SERPINB2 SOD2 STAT5A TNF* | | 60  [Methylene Chloride ↔ Breast Neoplasms \| CTD (ctdbase.org)](https://ctdbase.org/detail.go?type=relationship&chemAcc=D008752&diseaseAcc=MESH%3AD001943&view=reference) | **25 genes:** *AKT1 CTNNB1 CYP1A2 CYP1B1 CYP2E1 DAPK1 EGR1 ESR1 FOS GCLC GPX1 GSTT1 HMOX1 ID3 IFNG IL10 IL6 JUN JUNB MAP3K8 MMP1 NOS2 STAT5A TNF USP18* | 52  [Methylene Chloride ↔ Lung Neoplasms \| CTD (ctdbase.org)](https://ctdbase.org/detail.go?type=relationship&chemAcc=D008752&diseaseAcc=MESH%3AD008175&view=reference)  Has associated exposure references |
|  | | 1,2-Dichloropropane | | 78-87-5 | | **5 genes:** *H2AX KRT8 NDUFS3 TNF TP53BP1* | | 12  [propylene dichloride ↔ Breast Neoplasms \| CTD (ctdbase.org)](https://ctdbase.org/detail.go?type=relationship&chemAcc=C004765&diseaseAcc=MESH%3AD001943&view=reference) | **3 genes:** *CYP2E1 GSTM TNF* | 7  [propylene dichloride ↔ Lung Neoplasms \| CTD (ctdbase.org)](https://ctdbase.org/detail.go?type=relationship&chemAcc=C004765&diseaseAcc=MESH%3AD008175&view=reference) |
|  | | Trichloroethylene | | 79-01-6 | | **230 genes:** *ABCA8 ABCB1 ABCB1B ABCG2 ACACB ADAMTS1 AFP AGR2 AKAP12 AKT1 ANGPTL4 APC2 AR ARRDC3 ARTN ATG10 ATP6AP1L ATP7B AURKA BAG1 BARD1 BAX BCHE BCL2 BCL2A1 BGN BIRC5 C1QBP CAT CCL20 CCND1 CD40 CD74 CDA CDH1 CDH2 CENPF CFL1 CLDN4 CNR2 COMT COTL1 CPT1A CSF1 CSF2 CSF3 CTNNB1 CXCL12 CXCL8 CXCR4 CYP17A1 CYP1A1 CYP1B1 CYP24A1 CYP2B1 DDIT3 DEK DLL1DLL4 DNMT1 DNMT3A DNMT3B DPYD DYNC2H1 EDNRB EEF2 EGF EGFR ENO1 EPOR ERBB2 ERBB3 ESR1 ESR2 ESRRA EVL EZH2 FASN FGF10 FGF4 FGFR1 FGFR2 FHL2 FLACC1 FLT1 FOS FOXM1 FOXP3 FOXQ1 GJA1 GPNMB GRB7 GSK3B GSTP1 GUCY1A2 H2AX HADHB HAPLN4 HES1 HEY2 HEYL HHEX HIC1 HMMR HMOX1 HNRNPK HNRNPL HNRNPR HOXB13 HP HSP90AA1 HSPA1B IBSP IFNB1 IFNG IGF1 IGFBP5 IL10 IL1B IL24 IL6 ITSN2 JAG1 JUN KCNH1 KDR KIT KLHDC7A KLK10 KRT14 KRT5 KRT8 L3MBTL3 LBX1 LEF1 LEP LEPR LRRC37A LSP1 MAL MDM2 MDM4 MEIS1 MFGE8 MIR141 MIR222 MKI67 MME MMP2 MMP9 MST1 MTDH MTHFR MTOR MTR MYH9 NAT2 NCOR1 NFE2L2 NFKBIA NOS2 NOS3 NOTCH1 NOTCH2 NOTCH3 NQO1 NR2F6 NRCAM NRG1 NUDT17 OCLN PARP1 PDE2A PER3 PHGDH PPARGC1B PPM1D PRC1 PRSS46 PTEN PTGS1 PTGS2 RAD51 RAD51B RB1 RBM3 RELA RGS2 RPL31 RPS4X RPS8 RUNX2 RXRB SERPINB2 SETBP1 SFRP2 SIM1 SLC22A18 SLC28A1 SLC2A1 SLC2A2 SLC39A6 SLC5A5 SNAI1 SNAI2 SNX32 SOD2 SPP1 STARD8 STAT3 STC2 STMN1 STXBP4 SULT1A1 TFAP2A TFRC TGM2 THBS1 TNF TOX3 TP53 TP53BP2 TP73 TUBB3 UBD UBE2C VIM YAP1 YBX1 ZFP366* | | 95  [Trichloroethylene ↔ Breast Neoplasms \| CTD (ctdbase.org)](https://ctdbase.org/detail.go?type=relationship&chemAcc=D014241&diseaseAcc=MESH%3AD001943&view=reference)  Has associated exposure references | No direct evidence on marker/mechanism of gene interaction |  |
|  | |  | |  | |  | |  |  |  |
|  | | 1,1,2,2-Tetrachloroethane | | 79-34-5 | | No direct evidence on marker/mechanism of gene interaction | | - | No direct evidence on marker/mechanism of gene interaction | - |
|  | | 1,2-Dibromoethane | | 106-93-4 | | **5 genes:** *LEF1 NFE2L2 NQO1 TP53 TRP63* | | 8  [Ethylene Dibromide ↔ Breast Neoplasms \| CTD (ctdbase.org)](https://ctdbase.org/detail.go?type=relationship&chemAcc=D015946&diseaseAcc=MESH%3AD001943&view=reference) | **6 genes:** *ACSM1 CES1 CYP2E1 EPHX1 GSTT1 TP53* | 16  [Ethylene Dibromide ↔ Lung Neoplasms \| CTD (ctdbase.org)](https://ctdbase.org/detail.go?type=relationship&chemAcc=D015946&diseaseAcc=MESH%3AD008175&view=reference) |
|  | | 1,2-Dichloroethane | | 107-06-2 | | No direct evidence on marker/mechanism of gene interaction | | - | No direct evidence on marker/mechanism of gene interaction | - |
|  | | Tetrachloroethylene | | 127-18-4 | | **40 genes:** *ABCB1B ANGPTL4 APRT AREG BAX BCHE BCL2 BIRC5 BMPR2 CAT CPT1A CTU2 CYP17A1 CYP2B1 EEF2 EIF2S2 ERBB3 ESRRA FOS GPNMB GPX2 HAPLN4 IFNG JUN MIF MMP3 NQO1 NR2F6 NRG1 OCLN PHGDH RAD51B RARB RB1CC1 RCCD1 RPS6KB2 STC2 TNF TP53 UBD* | | 25  [Tetrachloroethylene ↔ Breast Neoplasms \| CTD (ctdbase.org)](https://ctdbase.org/detail.go?type=relationship&chemAcc=D013750&diseaseAcc=MESH%3AD001943&view=reference)  Has associated exposure references | No direct evidence on marker/mechanism of gene interaction | - |
|  | | Vinyl bromide | | 593-60-2 | | No direct evidence on marker/mechanism of gene interaction | | - | No direct evidence on marker/mechanism of gene interaction | - |
|  | | Polychlorinated biphenyl | | 1336-36-3 | | No direct evidence on marker/mechanism of gene interaction | | - | No direct evidence on marker/mechanism of gene interaction | - |
|  | | Tetrachlorodibenzodioxin | | 1746-01-6 | | **489 genes:** *ABCA8 ABCB1 ABCB1B ABCC1 ABCG2 ABL1 ACACB ACHE ACTA2 ACVR1 ADAM10 ADAM33 ADAMTS1 ADAR AFP AGR2 AHR AKAP12 AKT1 AKT2 ALDOA ALK ALKBH8 ANGPTL4 ANKRD34A APOBEC3B APRT AR ARAF AREG ARHGDIA ARID1A ARRDC3 ARTN ATG10 ATM ATP7B AURKA B4GAT1 BAG1 BAP1 BARD1 BAX BCAR3 BCHE BCL2 BCL2A1 BGN BIRC2 BIRC5 BMP2 BMP4 BMPR2 BRCA1 BRCA2 BRF1 BRIP1 C1QBP CADM1 CASP7 CASP8 CAT CAV1 CCL20 CCND1 CCNE1 CCNH CCT5 CD109 CD40 CD74 CDA CDH1 CDH2 CDH5 CDKN1B CDKN2A CENPF CFL1 CHEK1 CHEK2 CLDN1 CLDN4 CLIC1 CNR2 COL7A1 COMT COTL1 CPT1A CRHR1 CSF1 CSF1R CSF2 CSF3 CST6 CTNNB1 CTU1 CTU2 CUL5 CXCL12 CXCL2 CXCL3 CXCL8 CXCL9 CXCR4 CYP17A1 CYP19A1 CYP1A1 CYP1B1 CYP24A1 CYP2B1 CYP3A4 DAP3 DDIT3 DEK DEPP1 DES DHFR DIO3 DKK1 DLL1 DLL4 DNMT1 DNMT3A DNMT3B DPYD DSC3 DTX3 DYNC2H1 E2F1 EDNRB EEF1B2 EEF2 EFEMP1 EFNA1 EGF EGFR EIF2S2 EIF6 ELK3 ELP1 ELP3 EMSY ENO1 EP300 EPB41L3 EPHB4 EPOR ERBB2 ERBB3 ESR1 ESR2 ESRRA ETS2 ETV4 EVL EXO1 EZH2 F3 FABP7 FASN FBL FBXW7 FGD5 FGF10 FGFR1 FGFR2 FHL2 FKBPL FLACC1 FLNA FLT1 FN1 FOS FOXA1 FOXM1 FOXP3 FOXQ1 FST FTO GDF10 GJA1 GNAI2 GPER1 GPI GPNMB GPX1 GPX2 GPX4 GRB7 GRIK2 GSK3B GSTP1 GUCY1A2 GZMB H1-2 H19 H2AX H2BC12 H2BC4 H6PD HADHB HAPLN4 HES1 HEY1 HEY2 HEYL HHEX HIC1 HIF1A HMMR HMOX1 HNRNPK HNRNPL HNRNPR HOXB13 HOXB9 HOXD11 HP HPSE HRAS HRG HSP90AA1 HSPA1B IBSP ICAM5 IDO1 IFNB1 IFNG IGBP1 IGF1 IGF1R IGFBP5 IGFBP7 IL10 IL1B IL24 IL6 ITSN2 JAG1 JAG2 JMJD6 JUN KCNH1 KDR KIT KLHDC10 KLHDC7A KLK10 KRAS KRT14 KRT18 KRT5 KRT8 L3MBTL3 LAMTOR5 LBX1 LDHAL6B LDHB LEF1 LEP LEPR LGR6 LIMD2 LLGL2 LOXL2 LPAR1 LRRC3B LSP1 MACIR MAL MALAT1 MAN2C1 MAP2K7 MAP3K1 MDM2 MDM4 MECOM MED12 MED28 MEIS1 MTTL6 MFGE8 MIF MIR126 MIR141 MIR22 MIR29A MIR301A* *MKI67 MME MMP1 MMP14 MMP1A MMP2 MMP3* *MMP9 MRPL13 MRPL19 MRPL9 MRPS22 MRPS23 MRPS28 MRPS7 MST1 MT3 MTDH MTHFR MTOR MTR MYH9 NAT2 NCOA1 NCOA2 NCOA3 NCOR1 NDRG1 NDUFS3 NECTIN2 NFE2L2 NFKBIA NISCH NOP9 NOS2 NOS3 NOTCH1 NOTCH2 NOTCH3 NOTCH4 NQO1 NQO2 NR2F1 NR2F6 NRG1 NRIP1 NSD2 NSUN6 NUDT17 NUDT2 OCLN PABPC1 PAEP PAK1 PALB2 PARP1 PCBP1 PCDHGB6 PDE2A PDGFA PDPK1 PDZK1 PER3 PGR PHB1 PHGDH PIK3CA PIM1 PIN1 PLA2G4A PPARGC1B PPM1D PPP1R12B PRC1 PTEN PTGS1 PTGS2 PTPRD RAD51 RAD51B RAD51C RAD54L RAF1 RALYL RARA RARB RB1 RB1CC1 RBM3 RBP4 RCCD1 RECQL RELA REPS2 RGS2 RIBC2 RMND1 RNF115 RNF182 ROR1 RPL23A RPL31 RPLP2 RPS4X RPS6 RPS6KB2 RPS7 RPS8 RRAD RSPO3 RUNX2 RXRB SERPINB2 SERPINB5 SETD2 SFRP1 SFRP2 SFRP5 SHMT1 SIM1 SIRT1 SLC10A6 SLC16A3 SLC22A18 SLC28A1 SLC2A1 SLC2A10 SLC2A2 SLC2A5 SLC39A6 SLC5A5 SLCO1B1 SNAI1 SNAI2 SNCG SNX32 SOD2 SPATA18 SPP1 SRC SREBF2 STARD8 STAT3 STAT5A STC2 STMN1 STXBP4 SULT1A1 SYNE1 SYNJ2 TAFA4 TANK TBX3 TERT TFAP2A TFPI2 TFRC TGM2 THBS1 THEMIS2 TLE3 TMEM25 TNF TNFSF10 TNIP1 TOP2A TOX3 TP53 TP53BP1 TP53BP2 TP73 TRERF1 TRIM12A TRIM47 TRMT11 TRP53 TRP63 TUBB3 TXN TYMS UBD UBE2C UBLCP1 UMPS UPK1B VDR VEGFB VEGFC VIM VPS39 WNT10B WT1 WWOX XRCC2 XRCC3 YAP1 YBX1 ZEB1 ZEB2 ZNF365* | | 178  [Tetrachlorodibenzodioxin ↔ Breast Neoplasms \| CTD (ctdbase.org)](https://ctdbase.org/detail.go?type=relationship&chemAcc=D013749&diseaseAcc=MESH%3AD001943&view=reference)  Has associated exposure references | **250 genes:** *A2M ACE ACSM1 ACTB ADA ADAM28 AKT1 ALX4 ANK3 ANXA2 APC APOA1 APOC3 APOE ARHGEF5 AS3MT ATG101 ATOX1 AVPI1 AZGP1 BAP1 BCL2L1 BECN1 BHLHE41 BIRC5 BRAF BRCA2 CA12 CALML3 CASP8 CAV1 CCL18 CCN1 CCN2 CCND1 CCNG1 CD274 CDH13 CDKN1A CDKN1B CDKN1C CDKN2A CEACAM1 CES1 CES1F CHD4 CHEK2 CHRNA2 CHRNA3 CHRNA7 CHRNB4 CHST15 CLCA2 CLPTM1L CLTB COL6A1 COX17 CPE CRP CTNNB1 CXCL14 CYP1A2 CYP1B1 CYP24A1 CYP2A6 CYP2E1 DAB2IP DAPK1 DDR1 DNAI7 DNASE1L3 DNMT3A DOK1 DOK2 DOK3 DPYD EAF2 EEF2 EFEMP1 EFNB2 EGFR EGR1 EHMT2 EMX2 EPHX1 ERBB2 ERBB3 ERCC1 ERCC6 ERGIC3 ESR1 FAS FASLG FEN1 FGF9 FGFR1 FGFR2 FHIT FOS FOSB FOSL2 FOXM1 FOXP3 FUBP1 GATA6 GC GCLC GJA1 GJB1 GPX1 GPX3 GRB7 GSTM1 GSTP1 GSTP2 GSTT1 HES1 HEY1 HILPDA HMOX1 HRAS HTRA1 ID3 IDS IER2 IFNG IGBP1 IKBKG IL10 IL1B IL1R2 IL2 IL24 IL6 IRF1 IRF4 JAG1 JUN JUNB JUND KDR KRAS LECT2 LMNTD1 MAP2K7 MAP3K8 MAP4K4 MAPK1 MAPK14 MAPK3 MARCKS MCL1 MET MIR136 MIR193A MIR222 MIR30A MIR34B MIR34C MIR369 MIR98 MMP1 MMP10 MPO MPP1 MTHFR MYC MYO18B NFYA NOS2 NOTCH2 NOTCH3 NPPA NRG1 OGG1 PCNA PDCD4 PDLIM4 PGGT1B PIK3CA PON1 PPBP PPP2R1B PRDX1 PRDX6 PRKN PTEN PTGIS PTMA PYCARD RAD52 RAF1 RAMP2 RARB RASSF1 RCHY1 RIOX2 RNASET2 ROBO1 RTEL1 RUNX3 SECISBP2L SELENBP1 SELENOP SERPINA1 SERPING1 SFTPB SFTPD SIDT2 SLC22A18 SLC3A2 SLC7A5 SLCO1B3 SMARCC1 SMC2 SND1 SOX2 SOX30 SOX9 SPP1 SPRY2 STAT5A STIM1 STK11 STN1 TEP1 TERT TFRC TGFB1 TGFBR2 TLR4 TMEM45A TNF TP53 TP63 TP73 TRP53 TSC2 TSHR TTR TYMS TYRP1 UGT2B17 USP18 VHL WNT5A WT1 XPC ZNF595* | 59  [Tetrachlorodibenzodioxin ↔ Lung Neoplasms \| CTD (ctdbase.org)](https://ctdbase.org/detail.go?type=relationship&chemAcc=D013749&diseaseAcc=MESH%3AD008175&view=reference)  Has associated exposure references |
|  | |  | |  | |  | |  |  |  |
|  | |  | |  | |  | |  |  |  |
|  | |  | |  | |  | |  |  |  |
|  | |  | |  | |  | |  |  |  |
| **Hydrocarbon Group** | **Chemical Name** | | **CAS Number** | | **Breast Neoplasms Inference Network** | | **Inference Score & reference link** | | **Lung Neoplasms Inference Network** | **Inference Score & reference link** |
| Polyaromatic Hydrocarbon | Benzo(a)pyrene | | 50-32-8 | | **492 genes:** *ABCA8 ABCB1 ABCB1B ABCC1 ABCG2 ABL1 ACACB ACHE ACTA2 ACVR1 ADAM10 ADAM33 ADAMTS1 ADAR AFP AGR2 AHR AKAP12 AKT1 AKT2 ALDOA ALK ALKBH8 ANGPTL4 ANKRD34A APC2 APOBEC3A APOBEC3B APRT AR\|ARAF AREG ARF1 ARHGDIA ARRDC3 ARTN ATG10 ATM ATP6AP1L ATP7B AURKA BAG1 BARD1 BAX BCAR3 BCHE BCL2 BCL2A1 BGN BIRC2 BIRC5 BMP2 BMP4 BMPR2 BRCA1 BRCA2 BRF1 BRIP1 BTN3A2 C1QBP CADM1 CASP7 CASP8 CAT CAV1 CCL20 CCND1 CCNE1 CCNH CD109 CD40 CD74 CDA CDH1 CDH2 CDH5 CDKN1B CDKN2A CENPF CFL1 CHEK1 CHEK2 CLDN1 CLDN4 CLIC1 CLUL1 CNR2 COL7A1 COMT COTL1 CPT1A CRHR1 CSF1 CSF1R CSF2 CSF3 CST6 CTNNB1 CTU1 CUL5 CXCL12 CXCL2 CXCL3 CXCL8 CXCL9 CXCR4 CYP17A1 CYP19A1 CYP1A1 CYP1B1 CYP24A1 CYP2B1 CYP2D6 CYP3A4 DAP3 DDIT3 DEK DEPP1 DES DHFR DIO3 DKK1 DLL1 DLL4 DNMT1 DNMT3A DNMT3B DPYD DSC3 DTX3 DYNC2H1 E2F1 EDNRB EEF1B2 EEF2 EFEMP1 EFNA1 EGF EGFR EIF2S2 EIF6 ELK3 ELP1 ENO1 EPB41L3 EPHB4 EPOR ERBB2 ERBB3 ESR1 ESR2 ESRRA ETS2 ETV4 EVL EXO1 EZH2 F3 FABP7 FASN FBL FBXW7 FGD5 FGF10 FGF3 FGF4 FGFR1 FGFR2 FHL2 FKBPL FLACC1 FLNA FLT1 FN1 FOS FOXA1 FOXM1 FOXP3 FOXQ1 FST FTO GALNT16 GDF10 GJA1 GNAI2 GPER1 GPI GPNMB GPX1 GPX2 GPX4 GRB7 GRIK2 GSK3B GSTP1 GUCY1A2 H1-2 H19 H2AX H2BC12 H2BC4 H6PD HADHB HAPLN4 HES1 HEY1 HEY2 HEYL HHEX HIC1 HIF1A HMMR HMOX1 HNRNPK HNRNPL HNRNPR HOXB13 HOXB9 HOXD11 HP HPSE HRAS HRG HSP90AA1 HSPA1B IBSP ICAM5 IDO1 IFNG IGBP1 IGF1 IGF1R IGFBP5 IGFBP7 IL10 IL1B IL24 IL6 ITSN2 JAG1 JAG2 JUN KCNH1 KDR KIT KLHDC10 KLHDC7A KLK10 KMT2D KRAS KRT14 KRT18 KRT5 KRT8 L3MBTL3 LBX1 LDHB LEF1 LEP LEPR LGR6 LIMD2 LINC00671 LLGL1 LLGL2 LOXL2 LPAR1 LRRC3B LSP1 MACIR MAL MALAT1 MAN2C1 MAP2K7 MAP3K1 MDM2 MDM4 MECOM MED12 MED28 MEIS1 METTL6 MFGE8 MIF MIR10A MIR126 MIR127 MIR141 MIR152 MIR200B MIR200C MIR206 MIR222 MIR24-2 MIR29A MIR31HG MIR429 MIR506 MKI67 MME MMP1 MMP14 MMP1A MMP2 MMP3 MMP9 MRPL13 MRPL19 MRPS28 MST1 MT3 MTHFR MTOR MTR MYH9 NAT2 NCOA1 NCOA2 NCOA3 NCOR1 NDRG1 NECTIN2 NFE2L2 NFKBIA NMBR NOS2 NOS3 NOTCH1 NOTCH2 NOTCH3 NOTCH4 NQO1 NQO2 NR2F1 NR2F6 NRCAM NRG1 NRIP1 NSD2 NSUN6 OCLN PABPC1 PAEP PAK1 PALB2 PARP1 PCBP1 PCDHGB6 PDE2A PDGFA PDPK1 PDZK1 PER3 PGR PHGDH PIK3CA PIM1 PIN1 PLA2G4A PLEKHD1 PPARGC1B PPM1D PPP1R12B PRC1 PTEN PTGS1 PTGS2 PTHLH PTPRD RAD51* *RAD51B RAD51C RAD54L RAF1 RALYL RARA RARB RB1 RB1CC1 RBM3 RBP4 RCCD1 RELA REPS2 RGS2 RIBC2* *RIC8A RNF182 ROR1 RPL23A RPL31 RPLP2 RPS4X RPS6 RPS6KB2 RPS7 RPS8 RRAD RSPO3 RUNX2 RXRB SERPINB2 SERPINB5 SETBP1 SETD2 SFRP1 SFRP2 SFRP5 SHMT1 SIM1 SIRT1 SLC10A6 SLC16A3 SLC22A18 SLC28A1 SLC2A1 SLC2A10 SLC2A2 SLC2A5 SLC39A6 SLC5A5 SLCO1B1 SNAI1 SNAI2 SNCG SNX32 SOD2 SPATA18 SPP1 SRC SREBF2 STARD8 STAT3 STC2 STMN1 STXBP4 SULT1A1 SYNE1 TAFA4 TANK TBX3 TCL1B TERT TFAP2A TFPI2 TFRC TGM2 THBS1 THEMIS2 TLE3 TMEM25 TNF TNFSF10 TNIP1 TOP2A TOX3 TP53 TP53BP1 TP53BP2 TP73 TRERF1 TRIM47 TRP53 TRP63 TUBB3 TXN TYMS UBD UBE2C UBLCP1 UMPS UPK1B VDR VEGFB VEGFC VIM VPS39 WNT10B WT1 WWOX XRCC2 XRCC3 YAP1 ZC3H11A ZEB1 ZEB2 ZFP366 ZNF365 ZNF366 ZNF404 ZNF432 ZSWIM5* | | 132  [Benzo(a)pyrene ↔ Breast Neoplasms \| CTD (ctdbase.org)](https://ctdbase.org/detail.go?type=relationship&chemAcc=D001564&diseaseAcc=MESH%3AD001943&view=reference)  Has associated exposure references | | **259 genes:** *A2M ACE ACSM1 ACTB ADA ADAM28 AKT1 ALX4 ANK3 ANKRD18A ANKRD20A2P ANXA2 AOC4P APC APOA1 APOC3 APOE ARHGEF5 AS3MT ATOX1 AVPI1 AZGP1 BCL2L1 BECN1 BHLHE41 BIRC5 BRAF BRCA2 CA12 CALML3 CASP8 CAV1 CBR2 CCL18 CCN1 CCN2 CCND1 CCNG1 CD274 CDH13 CDKN1A CDKN1B CDKN1C CDKN2A CEACAM1 CES1 CES1F CHD4 CHEK2 CHRNA2 CHRNA3 CHRNA5 CHRNA7 CHRNB4 CHST15 CLCA2 CLPTM1L CLTB\|COL6A1 COX17 CPE CRP CTNNB1 CWH43 CXCL14 CYP1A2 CYP1B1 CYP24A1 CYP2A6 CYP2E1 DAB2IP DAPK1 DDR1 DNAI7 DNASE1L3 DNMT3A DOK1 DOK2 DOK3 DPYD EAF2 EEF2 EFEMP1 EFNB2 EGFR EGR1 EHMT2 EMX2 EPHX1 ERBB2 ERBB3 ERCC1 ERCC6 ERGIC3 ESR1 FAS FASLG FEN1 FGF9 FGFR1 FGFR2 FHIT FOS FOSB FOSL2 FOXM1 FOXP3 GAST GATA6 GC GCLC GJA1 GJB1 GPX1 GPX3 GRB7 GSTM1 GSTP1 GSTP2 GSTT1 HES1 HEY1 HILPDA HMOX1 HRAS HTRA1 ID3 IDS IER2 IFNG IGBP1 IKBKG IL10 IL1B IL1R2 IL2 IL24 IL6 IQSEC1 IRF1 IRF4 JAG1 JUN JUNB JUND KDR KRAS LECT2 LINC00115 MAP2K7 MAP3K8 MAP4K4 MAPK1 MAPK14 MAPK3 MARCKS MCL1 MET MIR10A MIR1246 MIR127 MIR136 MIR154 MIR222 MIR302D MIR34B MIR34C MIR410 MIR4435-2HG MIR487B MIR494 MIR98 MIRLET7BHG MMP1 MMP10 MPO MPP1 MTHFR MUC12 MUC16 MYC MYO18B NFYA NOS2 NOTCH2 NOTCH3 NPPA NRG1 OGG1 PCNA PDCD1 PDCD4 PDLIM4 PGGT1B PIK3CA PON1 PPBP PRDX1 PRDX6 PRKN PTEN PTGIS PTMA PYCARD RAD52 RAF1 RAMP2 RARB RASSF1 RCHY1 RNASET2 ROBO1 RTEL1 RUNX3 SECISBP2L SELENBP1 SELENOP SERPINA1 SERPING1 SFTPB SFTPD SIDT2 SLC22A18 SLC3A2 SLC7A5 SLCO1B3 SMARCC1 SMC2 SND1 SOX2 SOX30 SOX9 SPP1 STIM1 STK11 STN1 TEP1 TERT TFRC TGFB1 TGFBR2 TLR4 TMEM45A TNF TP53 TP63 TP73 TRP53 TSHR TTR TYMS TYRP1 UGT2B17 USP18 VHL WNT5A WT1 XPC* | 44  [Benzo(a)pyrene ↔ Lung Neoplasms \| CTD (ctdbase.org)](https://ctdbase.org/detail.go?type=relationship&chemAcc=D001564&diseaseAcc=MESH%3AD008175&view=reference)  Has associated exposure references |
|  | Dibenz(a,h)anthracene | | 53-70-3 | | No direct evidence on marker/mechanism of gene interaction | | - | | **34 genes:** *A2M APOC3 BCL2L1 BIRC5 CCN1 CCN2 CCND1 CCNG1 CDKN1A CPE CYP1A2 CYP1B1 CYP2E1 EFNB2 EGR1 EPHX1 ERBB2 FEN1 FGF9 GSTM1 GSTP1 GSTP2 HILPDA MAP4K4 MARCKS MCL1 MTHFR PCNA PPBP PTGIS RAD52 SOX9 SPP1 TP53* | 31  [1,2,5,6-dibenzanthracene ↔ Lung Neoplasms \| CTD (ctdbase.org)](https://ctdbase.org/detail.go?type=relationship&chemAcc=C026486&diseaseAcc=MESH%3AD008175&view=reference) |
|  | Benz(a)anthracene | | 56-55-3 | | No direct evidence on marker/mechanism of gene interaction | | - | | No direct evidence on marker/mechanism of gene interaction | - |
|  | Biphenyl | | 92-52-4 | | No direct evidence on marker/mechanism of gene interaction | | - | | No direct evidence on marker/mechanism of gene interaction | - |
|  | Anthracene | | 120-12-7 | | **12 genes:** *AHR AR BAX CCND1 COMT CXCL8 CYP19A1 CYP1A1 CYP1B1 ESR1 PTGS2 TNF* | | 38  [anthracene ↔ Breast Neoplasms \| CTD (ctdbase.org)](https://ctdbase.org/detail.go?type=relationship&chemAcc=C034020&diseaseAcc=MESH%3AD001943&view=reference)  Has associated exposure references | | No direct evidence on marker/mechanism of gene interaction | - |
|  | Benzo(e)pyrene | | 192-97-2 | | No direct evidence on marker/mechanism of gene interaction | | - | | No direct evidence on marker/mechanism of gene interaction | - |
|  | Benzo(b)fluoranthene | | 205-99-2 | | No direct evidence on marker/mechanism of gene interaction | | - | | **52 genes:** *ADA ANK3 APOC3 AVPI1 BCL2L1 BIRC5 CAV1 CCN1 CCND1 CCNG1 CDKN1A CDKN1C CDKN2A CLCA2 CLTB CYP1A2 CYP1B1 CYP2E1 DNMT3A DOK3 EGFR EGR1 EPHX1 ESR1 FGF9 GATA6 GC GJB1 GSTM1 GSTP1 GSTP2 HEY1 HILPDA HMOX1 IL1B IL1R2 IL6 KRAS LMNTD1 MAP4K4 MCL1 MTHFR MUC16 PCNA PTMA RTEL1 SFTPD SMC2 SPP1 TGFBR2 TP53 TRP53* | 38  [benzo(b)fluoranthene ↔ Lung Neoplasms \| CTD (ctdbase.org)](https://ctdbase.org/detail.go?type=relationship&chemAcc=C006703&diseaseAcc=MESH%3AD008175&view=reference)  Has associated exposure references |
|  | Benzo(k)fluoranthene | | 207-08-9 | | No direct evidence on marker/mechanism of gene interaction | | - | | **33 genes:** *BIRC5 CCND1 CES1 CHST15 CXCL14 CYP1A2 CYP1B1 EFNB2 ERBB2 ESR1 FGF9 FHIT FOS FOSL2 GCLC GSTM1 GSTP1 GSTP2 HILPDA HMOX1 IL1B IL1R2 JUN MAPK14 MMP1 PON1 PPBP SELENBP1 TFRC TGFB1 TMEM45A TP53 WNT5A* | 36  [benzo(k)fluoranthene ↔ Lung Neoplasms \| CTD (ctdbase.org)](https://ctdbase.org/detail.go?type=relationship&chemAcc=C022921&diseaseAcc=MESH%3AD008175&view=reference) |
|  | Chrysene | | 218-01-9 | | No direct evidence on marker/mechanism of gene interaction | | - | | **35 genes:** *ADA ANK3 BIRC5 CAV1 CCND1 CCNG1 CDH13 CDKN1A CYP1A2 CYP1B1 CYP2E1 EPHX1 ESR1 FGF9 FOS GJB1 GSTM1 GSTP1 HILPDA IL6 IQSEC1 JUN JUNB MAP4K4 MUC16 PTMA RASSF1 ROBO1 SLC3A2 SLC7A5 SOX2 SPP1 TNF TRP53 WT1* | 39  [chrysene ↔ Lung Neoplasms \| CTD (ctdbase.org)](https://ctdbase.org/detail.go?type=relationship&chemAcc=C031180&diseaseAcc=MESH%3AD008175&view=reference)  Has associated exposure references |
