# Supplementary material for: Mapping the influence of hydrocarbons mixture on molecular mechanisms, involved in breast and lung neoplasms: in silico toxicogenomic data-mining
Source: Genes Environ. 2024 Jul 9;46:15. doi: 10.1186/s41021-024-00310-y (PMC11232146; doi:10.1186/s41021-024-00310-y)
Supplement: Supplementary file 5 — Supplementary Material 5 [file 41021_2024_310_MOESM5_ESM.docx]

**Supplementary Table 3:** Genes interacted with most of the investigated hydrocarbons that are linked to breast or lung cancer development

| **Cancer** | **Number of common genes** | **Genes interacted with most of the investigated hydrocarbons^*^** |
| --- | --- | --- |
| Breast | 87 | *ABCB1B AKT1 ANGPTL4 AR ATP7B BAX* ***BIRC5*** *BCL2 BCL2A1 BCHE CAT* ***CCND1*** *CDH1 CDH2 CENPF COMT CNR2 CPT1A CTNNB1 CXCL8 CXCL12 CXCR4 CYP1A1 CYP17A1* ***CYP1B1*** *CYP2B1 DNMT1* ***DNMT3A*** *DNMT3B EEF2 ERBB3* ***ESR1*** *ESRRA EZH2 FASN* ***FOS*** *FOXA1 GPNMB GPX2* ***GSTP1*** *H2AX HAPLN4 HEYL HHEX HIC1 HMMR* ***HMOX1 HRAS*** *IFNG* ***IL1B*** ***IL6*** *IL10* ***JUN KRAS*** *KRT8 LEF1 MIR141 MMP9 MST1 NCOR1 NFE2L2 NFKBIA NOS2 NQO1 NR2F6 NRG1 OCLN PER3 PHGHD PPARGC1B PRC1 PTGS1 PTGS2 RAD51B RGS2 SERPINB2 SLC2A2 SLC39A6 SNAI1 SNAI2 SOD2 STC2 SULT1A1* ***TFRC*** ***TNF*** ***TP53*** *UBD* |
| Lung | 44 | *ANK3 APOC3 BCL2L1* ***BIRC5*** *CCN1 CCN2* ***CCND1*** *CCNG1 CDKN1A CDKN1C CYP1A2* ***CYP1B1*** *CYP2E1* ***DNMT3A*** *EGR1 EPHX1* ***ESR1*** *FGF9* ***FOS*** *GC GCLC GJB1 GSTM1* ***GSTP1*** *GSTP2 GSTT1 HILPDA* ***HMOX1 HRAS IL1B*** ***IL6*** ***JUN*** ***JUNB KRAS*** *MAP4K4 SELENBP1 SOX9 SPP1* ***TFRC*** *TGFB1* ***TNF*** ***TP53*** *TRP53 XPC* |

^*^Genes in bold—***BIRC5, CCND1, CYP1B1, DNMT3A, ESR1, FOS, GSTP1, HMOX1, HRAS, IL1B, IL6, JUN, KRAS, TFRC*, *TNF*, and *TP53***—are common genes in the development of both cancers that are affected by some of the investigated hydrocarbons.
