# Supplementary material for: Mapping the influence of hydrocarbons mixture on molecular mechanisms, involved in breast and lung neoplasms: in silico toxicogenomic data-mining
Source: Genes Environ. 2024 Jul 9;46:15. doi: 10.1186/s41021-024-00310-y (PMC11232146; doi:10.1186/s41021-024-00310-y)
Supplement: Supplementary file 6 — Supplementary Material 6 [file 41021_2024_310_MOESM6_ESM.docx]

**Supplementary Table 4:** CLUEGO result for breast neoplasm using KEGG, Reactome and WikiPathway databases

| **ID** | **Term** | **Ontology Source** | **GO Group** | **P-Values*** | | **Associated Genes Found** | | |
| --- | --- | --- | --- | --- | --- | --- | --- | --- |
|  |  |  |  | **Term** | **Group** | **No.** | **Name** | **% Associated** |
| WP:2873 | AHR pathway | WikiPathways_23.02.2022 | 13 | 7.26E-11 | 6.06E-11 | 10 | *BAX, CYP1A1, CYP1B1, IFNG, IL1B, JUN, NFE2L2, NQO1, SERPINB2, TNF* | 20.8 |
| WP:138 | Androgen receptor signaling pathway | WikiPathways_23.02.2022 | 19 | 3.34E-03 | 3.04E-07 | 6 | *AKT1, AR, CCND1, CTNNB1, JUN, NCOR1* | 6.6 |
| KEGG:04210 | Apoptosis | KEGG_25.05.2022 | 51 | 9.97E-09 | 5.01E-18 | 12 | *AKT1, BAX, BCL2, BCL2A1, BIRC5, FOS, HRAS, JUN, KRAS, NFKBIA, TNF, TP53* | 8.8 |
| KEGG:05224 | Breast cancer | KEGG_25.05.2022 | 27 | 2.47E-08 | 2.19E-12 | 12 | *BAX, CCND1, FOS, HRAS, KRAS, PGR, TP53* | 8.2 |
| KEGG:05204 | Chemical carcinogenesis | KEGG_25.05.2022 | 34 | 9.10E-03 | 4.42E-13 | 5 | *CYP1A1, CYP1B1, GSTP1, PTGS2, SULT1A1* | 7.3 |
| R-HSA:9710421 | DNA methylation | REACTOME_Pathways_25.05.2022 | 15 | 5.62E-02 | 4.97E-04 | 4 | *DNMT1, DNMT3A, DNMT3B, H2AX* | 6.2 |
| WP:697 | Estrogen metabolism | WikiPathways_23.02.2022 | 34 | 2.15E-05 | 4.42E-13 | 5 | *COMT, CYP1A1, CYP1B1, NQO1, SULT1A1* | 26.3 |
| WP:712 | Estrogen signaling pathway | WikiPathways_23.02.2022 | 28 | 5.80E-05 | 1.11E-08 | 5 | *AKT1, BCL2, ESR1, FOS, JUN* | 21.7 |
| R-HSA:9634638 | Estrogen-dependent nuclear events downstream of ESR-membrane signaling | REACTOME_Pathways_25.05.2022 | 17 | 2.36E-03 | 3.09E-09 | 4 | *AKT1, BCL2, CCND1, FOS* | 16.7 |
| KEGG:04216 | Ferroptosis | KEGG_25.05.2022 | 3 | 3.37E-02 | 1.53E-02 | 3 | *HMOX1, TFRC, TP53* | 7.3 |
| KEGG:05418 | Fluid shear stress and atherosclerosis | KEGG_25.05.2022 | 14 | 3.30E-05 | 2.32E-22 | 14 | *AKT1, BCL2, CTNNB1, FOS, GSTP1, HMOX1, IFNG, IL1B, JUN, MMP9, NFE2L2, NQO1, TNF, TP53* | 10.1 |
| WP:4495 | IL-10 anti-inflammatory signaling pathway | WikiPathways_23.02.2022 | 23 | 7.20E-03 | 2.12E-06 | 3 | *HMOX1, IL10, IL6* | 25 |
| KEGG:05417 | Lipid and atherosclerosis | KEGG_25.05.2022 | 58 | 3.37E-12 | 2.83E-13 | 17 | *AKT1, BAX, BCL2, CXCL8, CYP1A1, FOS, HRAS, IL1B, IL6, JUN, KRAS, MMP9, NFE2L2, NFKBIA, SOD2, TNF, TP53* | 8.0 |
| WP:1545 | miRNAs involved in DNA damage response | WikiPathways_23.02.2022 | 15 | 1.67E-01 | 4.97E-04 | 3 | *CCND1, H2AX, TP53* | 6.0 |
| KEGG:05206 | miRNAs in cancer | KEGG_25.05.2022 | 58 | 1.46E-07 | 2.83E-13 | 15 | *BCL2, CCND1, CYP1B1, DNMT1, DNMT3A, DNMT3B, ERBB3, EZH2, HMOX1, HRAS, KRAS, MIR141, MMP9, PTGS2, TP53* | 4.9 |
| WP:2884 | NRF2 pathway | WikiPathways_23.02.2022 | 26 | 6.31E-04 | 2.41E-19 | 8 | *GPX2, GSTP1, HMOX1, NFE2L2, NQO1, NRG1, SLC2A2, SLC39A6* | 5.5 |
| WP:2882 | Nuclear receptors meta-pathway | WikiPathways_23.02.2022 | 14 | 8.92E-19 | 2.32E-22 | 25 | *ANGPTL4, BAX, CCND1, CPT1A, CYP1A1, CYP1B1, ESR1, FASN, GPX2, GSTP1, HMOX1, IFNG, IL1B, JUN, NFE2L2, NQO1, NRG1, PTGS2, RGS2, SERPINB2, SLC2A2, SLC39A6, SNAI2, SULT1A1, TNF* | 7.76 |
| WP:408 | Oxidative stress response | WikiPathways_23.02.2022 | 26 | 3.35E-07 | 2.41E-19 | 7 | *CAT, CYP1A1, FOS, HMOX1, NFE2L2, NQO1, SOD2* | 20.6 |

^*^Corrected with Bonferroni step down
