# Supplementary material for: Mapping the influence of hydrocarbons mixture on molecular mechanisms, involved in breast and lung neoplasms: in silico toxicogenomic data-mining
Source: Genes Environ. 2024 Jul 9;46:15. doi: 10.1186/s41021-024-00310-y (PMC11232146; doi:10.1186/s41021-024-00310-y)
Supplement: Supplementary file 7 — Supplementary Material 7 [file 41021_2024_310_MOESM7_ESM.docx]

**Supplementary Table 5:** CLUEGO result for lung neoplasm using KEGG, Reactome and WikiPathway databases

| **ID** | **Term** | **Ontology Source** | **GO Group** | **P-Values*** | | **Associated Genes Found** | | |
| --- | --- | --- | --- | --- | --- | --- | --- | --- |
|  |  |  |  | **Term** | **Group** | **No.** | **Name** | **% Associated** |
| WP:2586 | AHR pathway | WikiPathways_23.02.2022 | 4 | 3.92E-10 | 2.48E-11 | 8 | *CDKN1A, CYP1A2, CYP1B1, ESR1, GCLC, HRAS, KRAS, TNF* | 16.7 |
| KEGG:04210 | Apoptosis | KEGG_25.05.2022 | 15 | 1.75E-06 | 1.13E-09 | 8 | *BCL2L1, BIRC5, FOS, HRAS, JUN, KRAS, TNF, TP53* | 5.9 |
| KEGG:05208 | Chemical carcinogenesis | KEGG_25.05.2022 | 11 | 3.57E-07 | 1.18E-10 | 7 | *CYP1A2, CYP1B1, CYP2E1, EPHX1, GSTM1, GSTP1, GSTT1* | 10.1 |
| KEGG:04216 | Ferroptosis | KEGG_25.05.2022 | 3 | 9.74E-04 | 5.55E-07 | 4 | *GCLC, HMOX1, TFRC, TP53* | 9.8 |
| KEGG:05418 | Fluid shear stress and atherosclerosis | KEGG_25.05.2022 | 17 | 8.42E-08 | 1.34E-09 | 9 | *FOS, GSTM1, GSTP1, GSTT1, HMOX1, IL1B, JUN, TNF, TP53* | 6.5 |
| WP:286 | IL-3 signaling pathway | WikiPathways_23.02.2022 | 8 | 7.03E-05 | 1.66E-08 | 5 | *BCL2L1, FOS, HRAS, JUN, TGFB1* | 10.2 |
| R-HSA:6785807 | IL-4 and IL-13 signaling | REACTOME_Pathways_25.05.2022 | 23 | 1.02E-13 | 2.16E-06 | 12 | *BCL2L1, BIRC5, CCND1, CDKN1A, FOS, HMOX1, IL1B, IL6, JUNB, TGFB1, TNF, TP53* | 11.1 |
| KEGG:04657 | IL-17 signaling pathway | KEGG_25.05.2022 | 17 | 1.28E-03 | 1.34E-09 | 5 | *FOS, IL1B, IL6, JUN, TNF* | 5.3 |
| KEGG:05417 | Lipid and atherosclerosis | KEGG_25.05.2022 | 17 | 3.66E-06 | 1.34E-09 | 9 | *BCL2L1, FOS, HRAS, IL1B, IL6, JUN, KRAS, TNF, TP53* | 4.2 |
| WP:3624 | Lung fibrosis | WikiPathways_23.02.2022 | 23 | 1.91E-07 | 2.16E-06 | 7 | *CCN2, HMOX1, IL1B, IL6, SPP1, TGFB1, TNF* | 11.1 |
| WP:1545 | miRNAs involved in DNA damage response | WikiPathways_23.02.2022 | 16 | 1.66E-02 | 5.48E-09 | 3 | *CCND1, CDKN1A, TP53* | 6.0 |
| WP:4255 | Non-small cell lung cancer | WikiPathways_23.02.2022 | 25 | 4.13E-04 | 1.14E-07 | 5 | *CCND1, CDKN1A, HRAS, KRAS, TP53* | 6.9 |
| WP:2884 | NRF2 pathway | WikiPathways_23.02.2022 | 1 | 7.80E-04 | 3.07E-14 | 6 | *EGR1, GCLC, GSTM1, GSTP1, HMOX1, TGFB1* | 4.1 |
| WP:2882 | Nuclear receptors meta-pathway | WikiPathways_23.02.2022 | 5 | 2.51E-13 | 3.03E-16 | 16 | *APOC3, CCND1, CDKN1C, CYP1A2, CYP1B1, EGR1, ESR1, GCLC, GSTM1, GSTP1, HMOX1, IL1B, JUN, JUNB, TGFB1, TNF* | 5.0 |
| WP:3941 | Oxidative damage response | WikiPathways_23.02.2022 | 4 | 1.22E-02 | 2.48E-11 | 3 | *CDKN1A, CDKN1C, TNF* | 7.3 |
| WP:408 | Oxidative stress response | WikiPathways_23.02.2022 | 2 | 4.97E-04 | 2.41E-12 | 4 | *FOS, GCLC, HMOX1, JUNB* | 11.8 |
| KEGG:04115 | p53 signaling pathway | KEGG_25.05.2022 | 12 | 4.39E-04 | 1.60E-09 | 5 | *BCL2L1, CCND1, CCNG1, CDKN1A, TP5* | 6.9 |
| WP:4658 | Small cell lung cancer | WikiPathways_23.02.2022 | 12 | 1.52E-03 | 1.60E-09 | 5 | *BCL2L1, CCND1, CDKN1A, CDKN1C, TP53* | 5.1 |
| R-HSA:2990846 | SUMOylation | REACTOME_Pathways_25.05.2022 | 1 | 2.53E-03 | 2.72E-05 | 5 | *BIRC5, DNMT3A, ESR1, TP53, XPC* | 3.2 |

^*^Corrected with Bonferroni step down
