# Supplementary material for: Mapping the influence of hydrocarbons mixture on molecular mechanisms, involved in breast and lung neoplasms: in silico toxicogenomic data-mining
Source: Genes Environ. 2024 Jul 9;46:15. doi: 10.1186/s41021-024-00310-y (PMC11232146; doi:10.1186/s41021-024-00310-y)
Supplement: Supplementary file 8 — Supplementary Material 8 [file 41021_2024_310_MOESM8_ESM.docx]

**Supplementary Table 6**: Genes connected to selected non-hydrocarbon carcinogens and linked to breast and lung neoplasms^*^

| **Chemical Name** | **CAS Number** | **Breast Neoplasms Inference Network** | **Inference Score & reference link** | **Lung Neoplasms Inference Network** | **Inference Score & reference link** |
| --- | --- | --- | --- | --- | --- |
| Acetaldehyde | 75-07-0 | **49 genes:** *ABL1 ACHE ACTA2 BAX BCL2 BMP2 CAT* ***CCND1*** *CSF CXCL8 CYP17A1 CYP19A1 DDIT3 DKK1 EEF2 EFNA1 EGF ERBB2 ETV4 FN1* ***FOS*** *FOXQ1 HES1 HHEX HIF1A IFNB1 IL10* ***IL1B IL6*** ***JUN*** *LEF1 LEP MECOM MMP1 MMP2 MMP9 MTOR MTR NFKBIA NOS2 OCLN PARP1 PTGS1 PTGS2 RELA RSPO3 SPP1* ***TFRC TNF*** | 94  https://ctdbase.org/detail.go?type=relationship&chemAcc=D000079&diseaseAcc=MESH%3AD001943&view=reference | **30 genes:** *CCN2* ***CCND1*** *CDKN1A CYP2E1 EEF2 EGR1 ERBB2* ***FOS*** *GCLC HES1 HILPDA IL10* ***IL1B IL6*** ***JUN*** *MAPK1 MAPK3 MCL1 MMP1 MPO MYC NOS2 PRK SPP1 SPRY2* ***TFRC*** *TGFB1 TGFBR2* ***TNF*** *WNT5A* | 57 https://ctdbase.org/detail.go?type=relationship&chemAcc=D000079&diseaseAcc=MESH%3AD008175&view=reference |
| Asbestos | 1332-21-4 | **42 genes:** *ATM BAP1 BAX BCL2 BRCA1 BRCA2 CD40 CDKN2A CSF1 CSF2 CSF3 CXCL12 CXCL2 CXCL8 EGFR EPB41L3 F3 FGF10 FN1* ***FOS*** ***GSTP1 HMOX1*** *IFNG IL10* ***IL6 JUN*** *MIF NAT2 NOS2 PALB2 PARP1 PTGS2 RARB RELA SPP1* ***TFRC TNF TP53*** *TP73 TRP53 TXN XRCC3* | 76 https://ctdbase.org/detail.go?type=relationship&chemAcc=D001194&diseaseAcc=MESH%3AD001943&view=reference | **41 genes:** *ANXA2 BAP1 BRCA2 CCN2 CDKN1A CDKN2A CES1 EGFR EPHX1 FAS FASLG FOS FOSB GSTM1 GSTP1 GSTT1 HMOX1 IFNG IL10 IL6 JUN MAPK1 MAPK14 MIRLET7BHG MPO NOS2 PRDX1 PTGIS PYCARD RARB RASSF1 SERPINA1 SPP1 TFRC TGFB1 TGFBR2 TNF TP53 TP73 TRP53 XPC* | 93 https://ctdbase.org/detail.go?type=relationship&chemAcc=D001194&diseaseAcc=MESH%3AD008175&view=reference |
| Bis(2-ethyl hexyl) phthalate | 117-81-7 | **260 genes:** *ABCB1 ABCB1B ABCG2 ACACB ACHE ACTA2 ADAMTS1 AFP AHR AKT1 AKT2 ALDOA ANGPTL4 AR ARHGDIA ARRDC3 ATM AURKA BAP1 BAX BCHE BCL2 BMP2 BMP4 BRCA1 BRCA2 BRIP1 BTN3A2 CADM1 CASP7 CASP8 CAT CAV1* ***CCND1*** *CCNE1 CCNH CD109 CD40 CD74 CDA CDH1 CDH2 CDKN1B CDKN2A CENPF CHEK1 CHEK2 CLDN1 CMC2 COL7A1 COMT COTL1 CPT1A CRHR1 CSF1R CSF2 CST6 CTNNB1 CXCL2 CXCL8 CXCR4 CYP17A1 CYP19A1 CYP1A1* ***CYP1B1*** *CYP24A1 CYP2D6 CYP3A4 DDIT3 DEPP1 DHFR DIO3 DLL1 DLL4 DNMT1 DNMT3B DPYD DTX3 EDNRB EEF2 EFNA1 EGF EGFR* *ENO1 EPB41L3 ERBB2 ERBB3* ***ESR1*** *ESR2 ESRRA ETV4 EZH2 FABP7 FASN \| FGF4 FGFR1 FGFR2 FHL2 FLACC1 FLNA FLT1 FN1* ***FOS*** *FOXA1 FOXQ1 FST FTO GALNT16 GDF10 GJA1 GPER1 GPX1 GPX4 GRB7 GRIK2 GSK3B* ***GSTP1*** *H2AX H6PD HADHB HES1 HEY1 HEY2 HEYL HIF1A* ***HMOX1*** *HNRNPK HP HSP90AA1 HSPA1B IDO1 IFNG IGF1 IGF1R IGFBP5 IGFBP7 IL10* ***IL1B IL6*** *JAG1 JAG2* ***JUN*** *KIT KRT14 KRT18 KRT5 KRT8 LDHB LEP LEPR LOXL2 LRRC3B MACIR MAL MDM2 MECOM MIR132 MIR141 MIR146A MIR152 MIR200B MIR200C MIR221 MIR222 MIR29A MIR345 MIR429 MIR489 MKI67 MME MMP14 MMP2 MMP9 MRPL9 MRPS28 MRPS7 MT3 MTOR MYH9 NCOA1 NDRG1 NDUFS3 NFE2L2 NOS2 NOS3 NOTCH1 NOTCH2 NOTCH3 NOTCH4 NQO1 NQO2 NRCAM NRG1 OCLN PARP1 PCBP1 PDE2A PDPK1 PDZK1 PER3 PGR PHGDH PIK3CA PPARGC1B PTEN PTGS1 PTGS2 PTHLH RAD51 RAD51B RAD54L RALYL RB1 RBM3 RBP4 RELA RGS2 RPL31 RPS4X RPS6 RUNX2 RXRB SERPINB2 SFRP2 SIRT1 SLC16A3 SLC2A1 SLC2A2 SLC5A5 SLCO1B1 SNAI1 SOD2 SPP1 SRC SREBF2 STC2 STXBP4 SULT1A1 SYNJ2 TAFA4 TERT TFPI2* ***TFRC*** *TGM2 THBS1 TLE3* ***TNF*** *TOP2A TOX3* ***TP53*** *TRERF1 TRP53 TUBB3 TXN VEGFB VEGFC VIM WNT10B WT1 YBX1* | 229 https://ctdbase.org/detail.go?type=relationship&chemAcc=D004051&diseaseAcc=MESH%3AD001943&view=reference | **139 genes:** *A2M ACSM1 ACTB AKT1 ANXA2 APOA1 APOC3 APOE AR ARHGEF5 BAP1 BCL2L1 BECN1 BHLHE41 BRCA2 CASP8 CAV1 CCN2* ***CCND1*** *CDH13 CDKN1A CDKN1B CDKN1C CDKN2A CEACAM1 CES1 CHD4 CHEK2 CHRNA2 CHRNA7 CHRNB4 CHST15 CLTB CRP CTNNB1 CYP1A2* ***CYP1B1*** *CYP24A1 CYP2A6 CYP2E1 DAPK1 DDR1 DOK3 DPYD EEF2 EGFR EGR1 EHMT2 EMX2 EPHX1 ERBB2 ERBB3 ERCC6* ***ESR1*** *FAS FGF9 FGFR1 FGFR2* ***FOS*** *FOSB FOSL2 GATA6 GCLC GJA1 GPX1 GRB7 GSTM1* ***GSTP1*** *HES1 HEY1 HILPDA* ***HMOX1*** *ID3 IER2 IFNG IKBKG IL10* ***IL1B IL6*** *JAG1* ***JUN*** *JUNB LECT2 MAPK1 MAPK14 MAPK3 MET MIR146A MIR193A MIR222 MIR224 MIR30A MIR31 MIR410 MIR494 MPO MPP1 MUC16 MYC NFE2L2 NOS2 NOTCH2 NOTCH3 NRG1 PCNA PIK3CA PRKN PTEN PYCARD RAMP2 RNASET2 ROBO1 SELENBP1 SELENOP SERPING1 SLC3A2 SLC7A5 SMC2 SOX2 SOX9 SPP1 TERT* ***TFRC*** *TGFB1 TGFBR2 TLR4* ***TNF*** ***TP53*** *TP63 TRP53 TSC2 TSHR TTR TYRP1 USP18 WT1 XPC* | 103 https://ctdbase.org/detail.go?type=relationship&chemAcc=D004051&diseaseAcc=MESH%3AD008175&view=reference |
| Cadmium | 7440-43-9 | *298 genes: ABCB1 ABCC1 ABCG2 ACACB ACHE ACTA2 AFP AHR AKAP12 AKT1 ALDOA ANGPTL4 APRT AR AREG ARF1 ARHGDIA ARID1A ATP6AP1L ATP7B AURKA BAG1 BAP1 BAX BCHE BCL2 BGN BIRC2 BMP2 BMP4 BRCA1 BRF1 CASP7 CASP8 CAT CAV1* ***CCND1*** *CCNE1 CCT5 CD109 CD74 CDA CDH1 CDH2 CDKN1B CDKN2A CENPF CFL1 CHEK1 CLDN1 CLIC1 CLUL1 COL7A1 COMT COTL1 CPT1A CSF1 CSF1R CSF2 CST6 CTNNB1 CXCL12 CXCL2 CXCL3 CXCL8 CXCL9 CXCR4 CYP17A1 CYP19A1 CYP1A1* ***CYP1B1*** *CYP24A1 DDIT3 DES DKK1 DLL1 DLL4 DNMT1 DNMT3B DYNC2H1 E2F1 EDNRB EEF1B2 EEF2 EFEMP1 EFNA1 EGF EGFR EIF2S2 ELP3 EMSY ENO1 EP300 EPB41L3 ERBB2 ERBB3* ***ESR1*** *ESR2 ETS2 EXO1 FASN FBL FBXW7 FGF4 FGFR1 FGFR2 FHL2 FLACC1 FLNA FLT1 FN1* ***FOS*** *FOXA1 FOXM1 FST GJA1 GPER1 GPI GPX1 GPX2 GPX4 GSK3B* ***GSTP1*** *GUCY1A2 H1-2 H19 H2AX H2BC12 H6PD HADHB HES1 HEYL HHEX HIF1A* ***HMOX1*** *HP HRG HSP90AA1 HSPA1B IBSP IDO1 IFNG IGF1 IGF1R IGFBP5 IGFBP7 IL10* ***IL1B IL6*** *JMJD6* ***JUN*** *KDR KIT KLK10 KMT2D KRT14 KRT5 KRT8 LAMTOR5 LEF1 LEP LEPR LIMD2 LOXL2 LPAR1 LRRC37A2 MALAT1 MAP2K7 MDM2 MECOM MEIS1 METTL6 MFGE8 MIF MIR101-2 MIR10B MIR132 MIR146A MIR152 MIR200C MIR206 MIR221 MIR222 MIR29A MIR489 MKI67 MME MMP1 MMP2 MMP3 MMP9 MRPL13 MRPS22 MRPS23 MST1 MT3 MTOR MYH9 NCOA1 NCOA2 NCOA3 NCOR1 NDUFS3 NFE2L2 NFKBIA NISCH NOP9 NOS2 NOS3 NOTCH1 NOTCH2 NOTCH3 NOTCH4 NQO1 NR2F6 NRG1 NRIP1 NSUN6 OCLN PAK1 PARP1 PDGFA PDPK1 PDZK1 PGR PHGDH PIK3CA PLA2G4A PPARGC1B PRC1 PTEN PTGS1 PTGS2 PTHLH RAD51 RAD51C RAF1 RARA RBM3 RBP4 RECQL RELA RGS2 RNF115 ROR1 RPLP2 RPS6 RRAD RSPO3 RUNX2 SERPINB2 SETD2 SFRP2 SHMT1 SIRT1 SLC16A3 SLC2A1 SLC2A10 SLC2A2 SLC2A5 SLC39A6 SLC5A5 SNAI1 SNAI2 SNCG SOD2 SPP1 SRC SREBF2 STAT3 STC2 STMN1 SYNE1 TFAP2A* ***TFRC*** *THBS1 TLE3* ***TNF*** *TNFSF10 TOP2* ***TP53*** *TP53BP2 TRERF1 TRIM47 TRP53 TUBB3 TXN TYMS UBE2C \| UMPS UPK1B VDR VEGFC VIM WNT10B WT1 ZEB1 ZNF404* | 223 https://ctdbase.org/detail.go?type=relationship&chemAcc=D002104&diseaseAcc=MESH%3AD001943&view=reference | **152 genes:** *A2M ACE AKT1 ALX4 ANXA2 APOA1 APOE AR ATG101 ATOX1 AVPI1 AZGP1 BAP1 BCL2L1 BECN1 BHLHE41 BRAF CA12 CALML CASP8 CAV1CBR2 CCN2* ***CCND1*** *CD274 CDKN1A CDKN1B CDKN1C CDKN2A CES1 CES1F CLCA2 COL6A1 COX17 CPE CRP CTNNB1 CXCL14* ***CYP1B1*** *CYP24A1 CYP2E1 DAB2IP DDR1 EEF2 EFEMP1 EFNB2 EGFR EGR1 EHMT2 EPHX1 ERBB2 ERBB3* ***ESR1*** *FAS FASLG FGFR1 FGFR2* ***FOS*** *FOSB FOSL2 FOXM1 FUBP1 GAST GC GCLC GJA1 GPX1 GPX3 GSTM1* ***GSTP1*** *GSTT1 HES1 HILPDA* ***HMOX1*** *HTRA1 IDS IFNG IKBKG IL10* ***IL1B*** *IL1R2 IL2* ***IL6 JUN*** *JUNB JUND KDR MAP2K7 MAPK1 MAPK14 MAPK3 MCL1 MET MIR146A MIR21 MIR222 MIR302D MIR30A MIR34B MIR410 MIR98 MMP1 MMP10 MPO MUC12 MYC NFE2L2 NOS2 NOTCH2 NOTCH3 NPPA NRG1 OGG1 PCNA PDLIM4 PGGT1B PIK3CA PON1 PPBP PPP2R1B PRDX1 PRDX6 PRKN PTEN PYCARD RAF1 RASSF1 RCHY1 SELENBP1 SELENOP SERPINA1 SERPING1 SFTPB SLC3A2 SOX9 SPP1 STK11 TEP1* ***TFRC*** *TGFB1 TGFBR2 TLR4* ***TNF*** ***TP53*** *TP63 TRP53 TTR TYMS USP18 VHL WNT5A WT1* | 90 https://ctdbase.org/detail.go?type=relationship&chemAcc=D002104&diseaseAcc=MESH%3AD008175&view=reference |
| Chromium (hexavalent) | 18540-29-9 | **154 genes:** *ABCB1B ABCC1 ABCG2 ACHE ACTA2 ADAMTS1 ADAR AHR AKT1 AR ARRDC3 ATG10 ATM BAX BCAR3 BCL2 BMP4 BRCA2 BRIP1 BTN3A2 C1QBP CASP7 CASP8 CAT CCL20* ***CCND1*** *CCNH CDA CDH1 CDH5 CDKN1B CDKN2A CHEK2 CMC2 COTL1 CTNNB1 CXCL8 CXCR4 CYP17A1 CYP19A1 CYP1A1* ***CYP1B1*** *DDIT3 DES DHFR DKK1 DPYD E2F1 EEF1B2 EEF2 EFNA1 EGFR EIF2S2 ELP1 EP300 ERBB2 ERBB3* ***ESR1*** *ESR2 ETV4 F3 FBL FN1* ***FOS*** *FST FTO GPNMB GPX1 GPX2 GPX4 GSK3B* ***GSTP1*** *GZMB H2AX HIF1A HMMR* ***HMOX1*** *HSP90AA1 IDO1 IFNG IGF1R* ***IL1B*** ***IL6 JUN*** *KIT KRT5 KRT8 LOXL2 LPAR1 MAP2K7 MIF MIR141 MIR200B MIR200C MMP2 MMP9 MRPL19 MRPS22 MRPS28 MRPS7 MTOR MYH9 NCOA1 NDUFS3 NFE2L2 NFKBIA NOP9 NOS2 NOTCH1 NQO1 OCLN PARP1 PDZK1 PHB1 PLA2G4A PTGS2 RAD51 RAD51B RAD51C RAD54L RB1 RBM3 RELA RGS2 RNF115 RPL23A RPL31 RPS6 RPS8 SHMT1 SIRT1 SLC2A1 SLC2A2 SLC2A5 SNAI1 SNAI2 SOD2 SRC SREBF2 STAT3 TERT* ***TFRC TNF*** *TNFSF10* ***TP53*** *TP53BP1 TRIM12A TRP53 TXN UMPS VDR VIM YBX1 ZEB1* | 151 https://ctdbase.org/detail.go?type=relationship&chemAcc=C074702&diseaseAcc=MESH%3AD001943&view=reference | ***86 genes:*** *ACE AKT1 APOA1 APOC3 AR AVPI1 BCL2L1 BECN1 BRCA2 CASP8* ***CCND1*** *CDKN1A CDKN1B CDKN2A CEACAM1 CHEK2 COL6A1 COX17 CRP CTNNB1 CWH43 CYP1A2* ***CYP1B1*** *DPYD EEF2 EFNB2 EGFR EGR1 ERBB2 ERBB3* ***ESR1*** *FAS FEN1 FGF9* ***FOS*** *GCLC GPX1 GPX3 GSTM1* ***GSTP1*** *HILPDA* ***HMOX1*** *IDS IER2 IFNG* ***IL1B*** *IL2* ***IL6*** *IRF1* ***JUN*** *JUNB LECT2 MAP2K7 MAPK1 MAPK14 MAPK3 MIR21 MIR302D MIR494 MIR607 MMP10 MYC NFE2L2 NOS2 OGG1 PCNA PDCD4 PRDX1 PRDX6 PRKN PTMA SERPINA1 SERPING1 SFTPB SIDT2 SMC2 SOX2 SOX9 TERT* ***TFRC*** *TGFBR2* ***TNF TP53*** *TRP53 USP18 WNT5A* | 78 https://ctdbase.org/detail.go?type=relationship&chemAcc=C074702&diseaseAcc=MESH%3AD008175&view=reference |

^*^Carcinogens other than hydrocarbons were selected from Supplementary Table 1 to gain insight on specificity of interacting genes related to breast and lung cancer. The 5 carcinogens interact with most of the 16 common genes, except *BIRC5, DNMT3A, HRAS* and *KRAS*, which are genes commonly interacted with the investigate hydrocarbons, namely carbon tetrachloride, 1,3-butadiene, TCDD, and BaP.
