# Supplementary material for: Mapping the influence of hydrocarbons mixture on molecular mechanisms, involved in breast and lung neoplasms: in silico toxicogenomic data-mining
Source: Genes Environ. 2024 Jul 9;46:15. doi: 10.1186/s41021-024-00310-y (PMC11232146; doi:10.1186/s41021-024-00310-y)
Supplement: Supplementary file 9 — Supplementary Material 9 [file 41021_2024_310_MOESM9_ESM.docx]

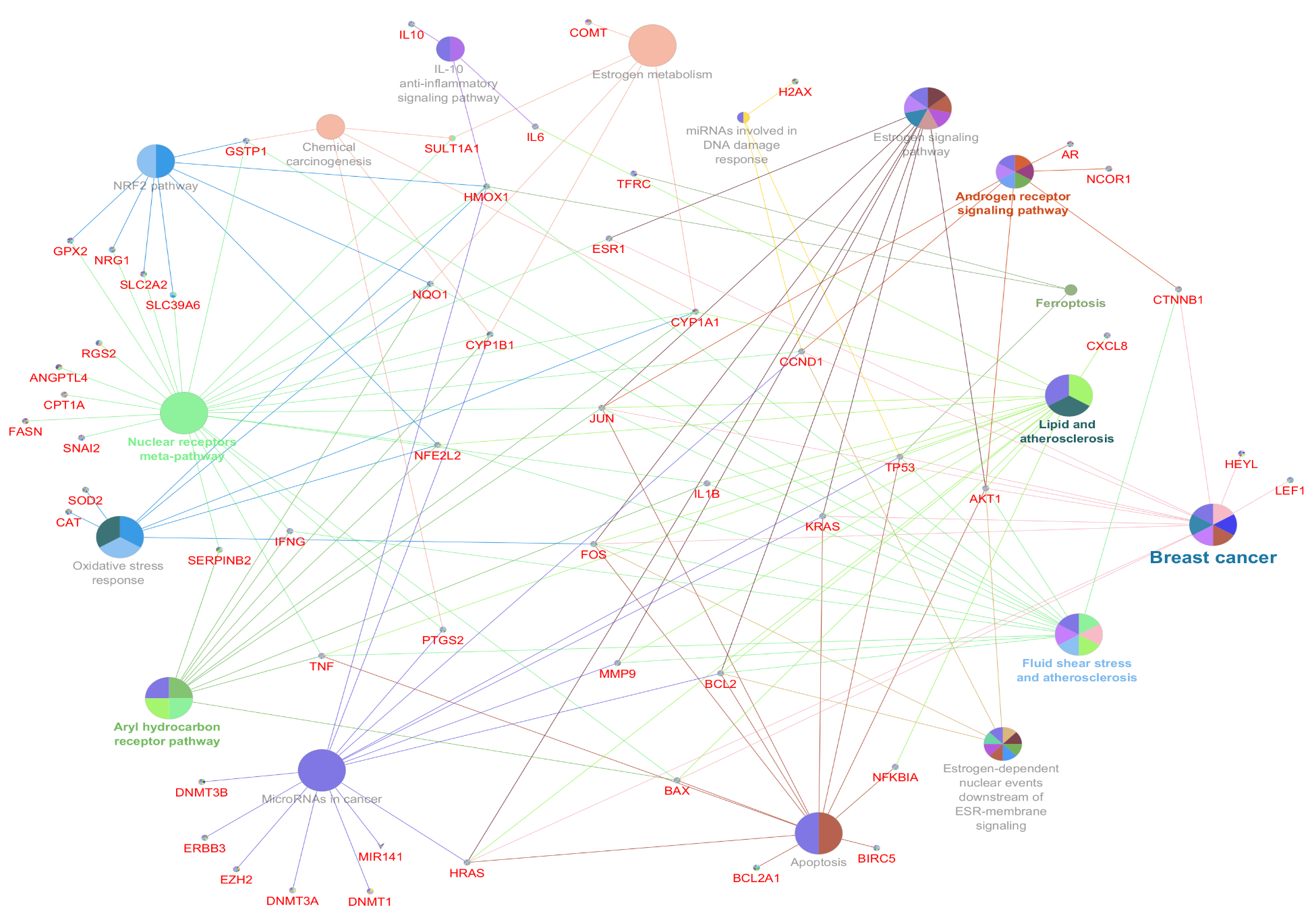


**Supplementary Figure 1:** Molecular pathways in the development of breast cancer that are linked to the investigated hydrocarbons extracted from the KEGG, Reactome and WikiPathways databases. The CLUEGO analysis parameters can be found in Supplementary Table 4.
